# The Discovery of New Deep-Sea Hydrothermal Vent Communities in the Southern Ocean and Implications for Biogeography

**DOI:** 10.1371/journal.pbio.1001234

**Published:** 2012-01-03

**Authors:** Alex D. Rogers, Paul A. Tyler, Douglas P. Connelly, Jon T. Copley, Rachael James, Robert D. Larter, Katrin Linse, Rachel A. Mills, Alfredo Naveira Garabato, Richard D. Pancost, David A. Pearce, Nicholas V. C. Polunin, Christopher R. German, Timothy Shank, Philipp H. Boersch-Supan, Belinda J. Alker, Alfred Aquilina, Sarah A. Bennett, Andrew Clarke, Robert J. J. Dinley, Alastair G. C. Graham, Darryl R. H. Green, Jeffrey A. Hawkes, Laura Hepburn, Ana Hilario, Veerle A. I. Huvenne, Leigh Marsh, Eva Ramirez-Llodra, William D. K. Reid, Christopher N. Roterman, Christopher J. Sweeting, Sven Thatje, Katrin Zwirglmaier

**Affiliations:** 1 Department of Zoology, University of Oxford, Oxford, United Kingdom; 2 Ocean and Earth Science, National Oceanography Centre, Southampton, University of Southampton, Southampton, United Kingdom; 3 Natural Environment Research Council, National Oceanography Centre, Southampton, Southampton, United Kingdom; 4 British Antarctic Survey, Cambridge, United Kingdom; 5 School of Chemistry, University of Bristol, Bristol, United Kingdom; 6 School of Marine Science and Technology, Newcastle University, Newcastle upon Tyne, United Kingdom; 7 Woods Hole Oceanographic Institution, Woods Hole, Massachusetts, United States of America; 8 Scottish Oceans Institute, University of St Andrews, St Andrews, United Kingdom; 9 Centro de Estudos do Ambiente e do Mar, Departmento Biologia, Universidade de Aveiro, Aveiro, Portugal; 10 Institut de Ciències del Mar, Consejo Superior de Investigaciones Científicas, Barcelona, Spain; University of California Davis, United States of America

## Abstract

Since the first discovery of deep-sea hydrothermal vents along the Galápagos Rift in 1977, numerous vent sites and endemic faunal assemblages have been found along mid-ocean ridges and back-arc basins at low to mid latitudes. These discoveries have suggested the existence of separate biogeographic provinces in the Atlantic and the North West Pacific, the existence of a province including the South West Pacific and Indian Ocean, and a separation of the North East Pacific, North East Pacific Rise, and South East Pacific Rise. The Southern Ocean is known to be a region of high deep-sea species diversity and centre of origin for the global deep-sea fauna. It has also been proposed as a gateway connecting hydrothermal vents in different oceans but is little explored because of extreme conditions. Since 2009 we have explored two segments of the East Scotia Ridge (ESR) in the Southern Ocean using a remotely operated vehicle. In each segment we located deep-sea hydrothermal vents hosting high-temperature black smokers up to 382.8°C and diffuse venting. The chemosynthetic ecosystems hosted by these vents are dominated by a new yeti crab (*Kiwa* n. sp.), stalked barnacles, limpets, peltospiroid gastropods, anemones, and a predatory sea star. Taxa abundant in vent ecosystems in other oceans, including polychaete worms (Siboglinidae), bathymodiolid mussels, and alvinocaridid shrimps, are absent from the ESR vents. These groups, except the Siboglinidae, possess planktotrophic larvae, rare in Antarctic marine invertebrates, suggesting that the environmental conditions of the Southern Ocean may act as a dispersal filter for vent taxa. Evidence from the distinctive fauna, the unique community structure, and multivariate analyses suggest that the Antarctic vent ecosystems represent a new vent biogeographic province. However, multivariate analyses of species present at the ESR and at other deep-sea hydrothermal vents globally indicate that vent biogeography is more complex than previously recognised.

## Abstract 2

A survey of Antarctic waters along the East Scotia Ridge in the Southern Ocean reveals a new vent biogeographic province among previously uncharacterized deep-sea hydrothermal vent communities.

## Author Summary

Deep-sea hydrothermal vents are mainly associated with seafloor spreading at mid-ocean ridges and in basins near volcanic island arcs. They host animals found nowhere else that derive their energy not from the sun but from bacterial oxidation of chemicals in the vent fluids, particularly hydrogen sulphide. Hydrothermal vents and their communities of organisms have become important models for understanding the origins and limits of life as well as evolution of island-like communities in the deep ocean. We describe the fauna associated with high-temperature hydrothermal vents on the East Scotia Ridge, Southern Ocean, to our knowledge the first to be discovered in Antarctic waters. These communities are dominated by a new species of yeti crab, stalked barnacles, limpets and snails, sea anemones, and a predatory seven-armed starfish. Animals commonly found in hydrothermal vents of the Pacific, Atlantic, and Indian Oceans, including giant *Riftia* tubeworms, annelid worms, vent mussels, vent crabs, and vent shrimps, were not present at the Southern Ocean vents. These discoveries suggest that the environmental conditions of the Southern Ocean may act as a barrier to some vent animals and that the East Scotia Ridge communities form a new biogeographic province with a unique species composition and structure.

## Introduction

The discovery of hydrothermal vents along the Galápagos Ridge in 1977 [1] led to the identification of chemoautotrophic symbiosis [2] and forced marine biologists to reassess the contribution chemosynthesis makes to marine primary production, particularly in the deep sea, where it supports a high biomass in an otherwise food-limited ecosystem. The existence of life in the extremely harsh conditions of hydrothermal vents has stimulated an increasing research effort on the diversity, ecology, and physiology of vent organisms, as well as new avenues of research into the origins of life on Earth [3] and even into the occurrence of life elsewhere within and outside the solar system. Because of the characteristics of hydrothermal vent communities—in particular the high levels of species endemism, their constraint to discrete habitats separated at different spatial scales and by geological/environmental barriers, their global distribution, and their historical coupling to plate tectonics—they are regarded as unique ecosystems. In particular, ecologists recognise that the unusual characteristics of deep-sea vents compared to other deep-sea habitats, coupled with the ephemeral nature of hydrothermal circulation, have probably had important implications for the composition, diversity, and biogeography of their communities and the dispersal and genetic population structure of vent species [4]–[6].

**Figure 1:**
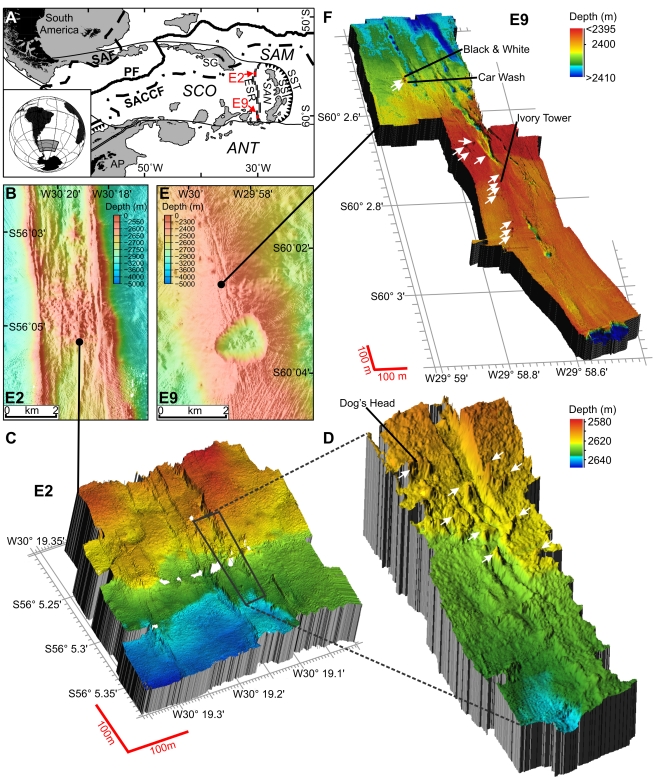
Maps of the position and geophysical setting of the ESR vents. (A) The Scotia Sea showing the ESR in relation to the Scotia Plate (SCO), South Sandwich Plate (SAN), South American Plate (SAM), the Antarctic Plate (ANT), the Antarctic Peninsula (AP), and the South Sandwich Trench (SST). Oceanographic features shown include the Polar Front (PF), the Sub-Antarctic Front (SAF), and the southern Antarctic Circumpolar Current Front (SACCF). The sites E2 and E9 are indicated by red arrows. (B) Ship-based swath bathymetry of the vent sites at E2 showing the axial summit graben. The black circle indicates the sites of main venting. (C and D) ROV-based 3-D swath bathymetry of E2 (C) and high-resolution swath bathymetry of the major steep-sided fissure that runs north–south through the centre of the site, between longitude 30° 19.10′W and 30° 19.15′W (D). Dog's Head vent site is indicated. White arrows indicate vent sites not mentioned in text. (E) Ship-based swath bathymetry of the vent sites at E9 showing the axial fissures and the collapsed crater called the Devil's Punchbowl. The black spot indicates the sites of main venting. (F) ROV-based 3-D swath bathymetry of the vent sites at E9. The vent sites Ivory Tower, Car Wash, and Black and White are indicated. Other vent sites are indicated by white arrows.

Several decades of exploration have resulted in the detection of numerous vent sites and faunal assemblages at many mid-ocean ridges and back-arc basins. These discoveries have resulted in an apparent global biogeography of vent organisms with separate provinces in the East Pacific, the North East Pacific, West Pacific back-arc basins, the shallow and deep Atlantic, and the Indian Ocean [7], although a more recent analysis has proposed a single province for the Atlantic, a single province for the North West Pacific, a single province for the South West Pacific and Indian Ocean, and a biogeographic separation of the North East Pacific, North East Pacific Rise, and South East Pacific Rise [8]. These biogeographic provinces are based on sampling undertaken by human-occupied vehicles and remotely operated vehicles (ROVs), and for the most part lie within the tropics and sub-tropics, where deep submergence operations are less limited by prevailing sea conditions than at high latitudes [6],[9]. Weather conditions have constrained the discovery of hydrothermal vents at high latitudes, although there is evidence from water column plumes that vents occur in the Arctic along the Gakkel Ridge [10], the Mohn Ridge, [11] and the Arctic Mid-Ocean Ridge [12], and in the Southern Ocean, in Antarctica, along the East Scotia Ridge (ESR), in the Scotia Sea [13], in the Bransfield Strait, west of the northern Antarctic Peninsula [14],[15], and along the Pacific-Antarctic Ridge [16]. In the Arctic, animal communities have been described at deep-sea hydrothermal vents on the Mohn and Arctic Mid-Ocean Ridges, although only the latter appears to host a high biomass of vent-endemic fauna [11],[12]. Here we show, to our knowledge for the first time, the presence of black smokers, diffuse venting, and associated chemosynthetically driven ecosystems along the ESR, a geographically isolated back-arc spreading centre in the Atlantic sector of the Southern Ocean, Antarctica (Figure 1A). Based on biological observations we also present a re-analysis of the global biogeography of the deep-sea hydrothermal vent fauna, including that of the Antarctic hydrothermal vents.

The Scotia Sea is defined by a loop of shallow banks and islands, known as the Scotia Arc, that extends eastwards from Cape Horn, south of the Falkland Islands (Burdwood Bank, Shag Rocks, and South Georgia), then southwards along the South Sandwich Arc, and westwards along the South Scotia Ridge, including the South Orkney Islands, to the tip of the Antarctic Peninsula near Elephant Island. The western boundary is formed by the Shackleton Fracture Zone. With the exception of these peripheral ridges, the ESR (Figure 1A) and various shallow banks (e.g., Pirie Bank, Bruce Bank), much of the Scotia Sea extends to depths in excess of 3,000 m. West of the ESR, the floor of the Scotia Sea forms part of the Scotia Plate. To the east of the ESR lies the small South Sandwich Plate, beneath which the South American Plate is being subducted at the South Sandwich Trench. To the north, the Scotia Plate abuts the South American Plate at the North Scotia Ridge, while to the south is the Antarctic Plate boundary at the South Scotia Ridge. Both of these are strike-slip plate boundaries [17]. The ESR is ∼500 km long, and spreading was initiated more than 15 million years ago (Mya) [18] and is presently proceeding at an average full spreading rate of ∼70 mm y^−1^. The ESR consists of nine second-order ridge segments (E1 to E9), separated by non-transform discontinuities [19]. E3 to E8 have well-developed deep rift valleys, but E2 and E9 are characterised by smooth volcanic highs, typical of faster-spreading mid-ocean ridges. An axial magma chamber is known to underlie segment E2 [20], and another is suspected to underlie segment E9 [21]. The southern end of segment E9 is curved to the east because of changes in the stress field as the strike-slip faults separating the South Sandwich and Scotia plates from the Antarctic plate are approached.

The first evidence of hydrothermal activity along the ESR was from data obtained by a light-scattering sensor attached to the Towed Ocean Bottom Instrument (TOBI), a deep-towed sonar system, during a geophysical mapping survey along the ESR in 1999 [13]. Additional evidence was obtained from conductivity–temperature–depth (CTD) profiles and manganese anomalies in water samples collected at depth during that survey. In the austral summer of 2009 we conducted a survey of segments E2 and E9 using a CTD sensor that was continuously raised and lowered in the water column (“tow-yo”), with attached light-scattering sensor and redox potential (Eh) sensors to track hydrothermal plumes and locate potential vent sites to within 100 to 500 m. We then used a lowered camera system, Seabed High Resolution Imaging Platform (SHRIMP), with down-looking and oblique video cameras to survey the seafloor in as systematic a fashion as possible. At E2 we located black smoker chimneys, as well as observing associated fauna, and at E9 we found considerable evidence of diffuse hydrothermal venting, with anemones and stalked barnacles being the dominant megafauna. Because SHRIMP is controllable only in the vertical plane, we withdrew it from the vent sites to prevent unnecessary damage, in accordance with InterRidge guidelines [22]. In the austral summer of 2010 we returned with the ROV *Isis* and conducted a full and systematic survey of the previously located vent sites at E2 and E9. This was supplemented by additional video analysis using SHRIMP in the austral summer of 2011.

## Results

### Hydrothermal Setting of Vents at E2 and E9

**Figure 2:**
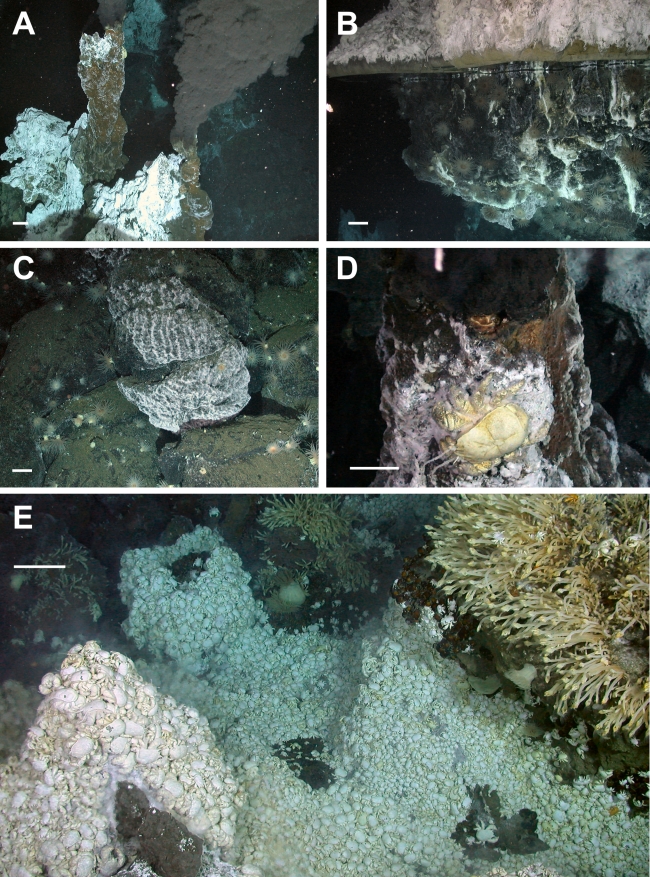
Photographs of vents and associated biological communities. (A) Active black smoker chimneys at E2 (Dive 128, 2,602 m depth). (B) Vent flange at E2 with trapped high-temperature reflective hydrothermal fluid (Dive 129, 2,621 m depth). (C) Microbial mat covering rock surfaces on vent periphery at E2 (Dive 134, 2,604 m depth). (D) Active vent chimney at E9 supporting the new species of the anomuran crab *Kiwa*. (Dive 144, 2,396 m depth). (E) Dense mass of the anomuran crab *Kiwa* n. sp. at E9 with the stalked barnacle cf. *Vulcanolepas* attached to nearby chimney (Dive 138, 2,397 m depth). Scale bars: 10 cm for foreground.

The vent sites at E2 lie just south of the segment axial high (called the Mermaid's Purse [20]), between 56° 5.2′ and 56° 5.4′ S and between 30° 19′ and 30° 19.35′W at ∼2,600 m depth (Figure 1B). Prominent north–south structural fabric to the seafloor defines a series of staircased, terraced features that are divided by west-facing scarps (Figure 1C and 1D). A major steep-sided fissure runs north–south through the centre of the site, between longitude 30° 19.10′W and 30° 19.15′W (Figure 1D). The fissure is filled in places by lobes of pillow basalts, and the main hydrothermal vents are located at the intersection between this main fissure and a west–east striking fault or scarp, consistent with the expected location of active venting on back-arc spreading ridges. Relict (extinct) and actively venting chimneys are resolvable in the high-resolution multibeam bathymetry obtained by the ROV *Isis*, clustered in a band running approximately northwest–southeast. Numerous volcanic cones and small volcanic craters are also apparent around the vent field. Chimneys of variable morphology were up to 15 m tall and venting clear fluid with a maximum measured temperature of 352.6°C, which formed focused black smokers on contact with cold seawater (Figure 2A). Some of the chimneys have expanded tops with hot vent fluid (>300°C) emanating from the underside (Figure 2B), similar to the flanges found at North East Pacific vents [23]. Diffuse vent flow was observed at a variety of locations, with temperatures varying from 3.5 to 19.9°C, compared with a background temperature of ∼0.0°C. Around the periphery of the active high-temperature vents and diffuse flow sites are microbial mats that form a halo around the venting area at E2 (Figure 2C).

The vent sites at E9 are situated between 60° 02.5′ and 60° 03.00′S and between 29° 59′ and 29° 58.6′W, at ∼2,400 m depth, amongst relatively flat sheet lavas to the north of a major collapse crater (named the Devil's Punchbowl; Figure 1E). The ridge axis is heavily crevassed and fissured, with numerous collapse features, lava drain-back features, and broken pillow lava ridges. Major fissures run north-northwest–south-southeast through the site, breaking up an otherwise flat and unvaried terrain (Figure 1F). Topographic highs in the centre of the study site are possibly dead magma domes, with no hydrothermal activity around these sites. Most active venting appears to lie along one of the smaller fissures, west of a main north–south trending feature. Diffuse flow and black smokers line the feature intermittently, but activity becomes reduced and dies away farther south, towards the “punchbowl” itself. The chimneys were either emitting high-temperature fluids with a maximum temperature of 382.8°C (Ivory Tower; Figure 1F) or had lower temperature diffuse flow between 5 and 19.9°C (Car Wash vent; Figure 1F). Low-temperature diffuse flow was associated with fissures and fine cracks in the sheet lava; the background temperature at E9 varied from −0.11°C to −1.3°C.

### Chemical and Physical Characteristics of E2 and E9

A summary of the preliminary chemical and physical data from the vents on both E2 and E9 north and south is given in Table 1. This table also includes data for the closest known vent sites to the ESR in the Atlantic, Indian, and Pacific Oceans, as well as from other hydrothermal vents associated with back-arc basins [24]–[28]. The chemical composition of fluids from E2 is distinct from that at E9, and within E9 there are notable differences in the vent fluid chemistry between vents in the northern part of the site and those in the southern part (Table 1). The chloride (Cl) concentration of fluids from E2 is similar to that of seawater, whereas fluids from E9 have very low levels of Cl and, as a consequence, they have lower concentrations of the major cations such as sodium, and higher concentrations of volatiles including hydrogen sulphide (H_2_S). This has the potential to impact the energy available for microbial populations at the vent sites, with volatile-dominated systems having higher hydrogen sulphide and hence higher microbial populations [29].

**Table 1:** Chemical composition of the vent fluid end-member at E2 and E9 vent fields.

Region	Site	Maximum Temperature (°C)	[Cl−] (mM)	pH	H_2_S (mM)	Na (mmol kg^−1^)	Si (mmol kg^−1^)
**ESR**	E2 (this study)	353	531	2.9	7.0	420	19
	E9N (this study)	383	98	3.4	9.5	96	8
	E9S (this study)	351	179	3.2	13.6	169	163
**Mid-Ocean Ridges**							
Atlantic Ocean	Nibelungen [26]	372	567	2.9	1.1	449	13.7
Indian Ocean	Kairei [25]	360	587	5.23		531	
Pacific Ocean	South East Pacific Rise [24]	340	190	3	8.6	125	10.6
**Back-Arc Basins**	Lau Basin [27]	334	650–800	2		520–615	14
	Pacmanus [28]	341	625	2.6	6.3	495	17.8
**Seawater**			541	7.9		464	0.18

Data from the Nibelungen vent field on the Mid-Atlantic Ridge [26], Kairei on the Central Indian Ridge [25], the 17.5°S site on the South East Pacific Rise [24], and sites in the Lau and Pacmanus back-arc basins [27],[28] are provided for comparison. These represent the closest known mid-ocean ridge vent sites to E2 and E9 and geologically comparable back-arc basin sites.

Initial analysis of the data from the seafloor-mounted acoustic doppler current profiler, deployed at E2 and E9 for 7 d each, suggests a semidiurnal north–south tidal flow with velocities between 50 and 100 mm s^−1^ in the bottom 50 m at E2. The flow in the bottom 50 m at E9 is more complicated, with an underlying semidiurnal tidal flow up to 100 mm s^−1^ plus an asymmetric west–east–west flow of ∼50 mm s^−1^.

### Microbial and Faunal Composition and Distribution at E2 and E9

Examination of 16S rDNA clone libraries from water samples taken within the buoyant vent plumes over E2 and E9 show a highly similar composition of the microbial communities at both sites. Proteobacteria make up 70% of the bacterial community at E2 (66% at E9). Within the proteobacteria, gammaproteobacteria are the dominant group (58% and 55% at E2 and E9, respectively). More than half of the gammaproteobacterial sequences (59% at E2 and 58% at E9) show high similarity (>99%) to bacterial endo- and epi-symbionts of hydrothermal vent fauna from elsewhere. Within the alphaproteobacteria, more than 90% of the sequences fall within the SAR11 clade, a group of ubiquitous heterotrophic bacteria found throughout the oceans. Other numerically abundant sequences in the clone libraries are closely related to Bacteroidetes (12% at E2 and 13% at E9) and Deferribacterales (11% at E2 and 12% at E9). Sequences for the bacterial clone libraries have been deposited in GenBank (http://www.ncbi.nlm.nih.gov/genbank/; accession numbers JN562472–JN562714).

At E2 and E9, the fauna is visually dominated by extensive dense aggregations of a new species of yeti crab, *Kiwa* n. sp. (Figure 2D and 2E). This species shows sequence divergences for mitochondrial 16S rDNA and nuclear 18S and 28S rDNA of 6.45%, 0.49%, and 1.8%, respectively, when compared with *K. hirsuta* from the Pacific-Antarctic Ridge (GenBank accession numbers JN628249, JN628250, JN628251). This variation is within the range of congeneric species comparisons for the Anomura [30], and a phylogenetic analysis, using Bayesian inference, of anomuran taxa, indicates that *Kiwa* n. sp. is the sister taxon of *K. hirsuta* (Figure S1). Using known substitution rates from geminate species pairs of anomuran crustaceans from either side of the Isthmus of Panama, the 16S data suggest a putative divergence between *K. hirsuta* and *Kiwa* n. sp. from the ESR at ∼12.2 Mya (0.53% per million years [31]), although such a preliminary date of divergence is subject to a high level of error. The new species of *Kiwa* from the ESR has dense mats of two distinct types of setae covering the ventral surface of the body, in contrast to *K. hirsuta*, which has sparse long setae on the ventral surface and a dense covering of long setae on the pereopods and particularly the chelipeds [9]. Filamentous bacteria were observed attached to the setae, as also seen in *K. hirsuta* [31]. Macpherson et al. [9] suggested that *K. hirsuta* is omnivorous, following observations of individuals consuming damaged mussels. However, the presence of sulphur-oxidising bacteria on the setae of this species [32] suggests that *K. hirsuta* may harvest bacteria as a nutritional source [9], and if this is the case, *Kiwa* n. sp. from E2 and E9 may also utilise epibiotic bacteria in the same way. At E2 dense aggregations of crabs may be found adjacent to and on chimneys, with large individuals closely associated with the vent orifice. At E9 *Kiwa* n. sp. was more abundant than at E2, completely covering the seabed in some areas and reaching densities of 600 m^−2^ (Figure 2E). At some sites this species formed multiple layer aggregations. The distribution of sexes appears to be influenced by distance from vent sources, possibly determined by temperature or vent fluid composition. Males were found closest to vent orifices (Figure 2D), and non-berried females adjacent to the vent but in cooler waters. Berried females and juveniles were associated with low-temperature flow, ∼5°C (as on Car Wash), and at the periphery of vent influence. They had considerably fewer filamentous bacteria on their setae than crabs near or on the chimneys, suggesting that the bacteria rely on the higher temperatures and chemistry in the immediate vicinity of the vent orifice for optimal growth.

**Figure 3:**
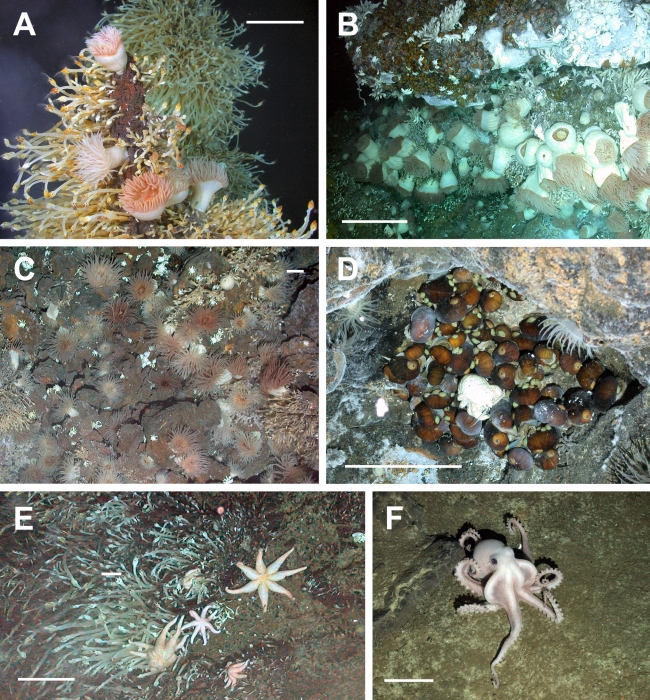
Photographs of the ESR vent fauna. (A) Actinostolid sea anemones surrounded by cf. *Vulcanolepas* on a chimney with diffuse hydrothermal venting at E9 (Dive 138, 2,396 m depth). (B) Dense field of actinostolid sea anemones along with peltospiroid gastropods (Dive 140, 2,394 m depth). (C) Anemone field at E9 with juvenile *Kiwa* n. sp. interspersed (Dive 139, 2,398 m depth). (D) Undescribed peltospiroid gastropod at E2 surrounding single *Kiwa* n. sp. and partially covered by *Lepetodrilus* n. sp. The pycnogonid cf. *Sericosura* is at the bottom right of the image (Dive 132, 2,608 m depth). (E) An undescribed seven-arm sea star predatory on the stalked barnacles cf. *Vulcanolepas* at E9 (Dive 139, 2,402 m depth). (F) Unidentified octopus at E9 (Dive 144, 2,394 m depth). Scale bars: 10 cm for foreground.

**Figure 4:**
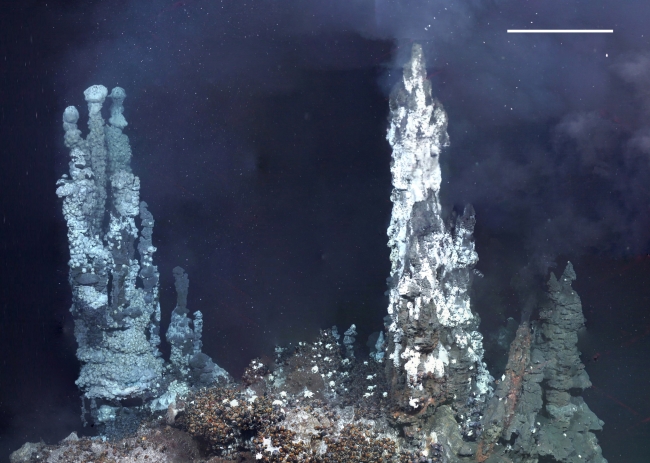
Collage of frame grabs of high-definition video to show fauna dispersion on the E9 vent site Ivory Tower. The vertical chimneys are covered with the anomuran *Kiwa* n. sp., and the area between the chimneys is occupied primarily by an undescribed peltospiroid gastropod (Dive 142, 2,398 m depth, ROV heading 090°). Scale bar: 1 m for foreground. Collage created by L. M.

Additional common fauna at the sites (Table 2) includes at least five morphospecies of sea anemone, three of which are found in diffuse flow associated with chimneys or sheet and pillow lavas in densities of up to ∼70 m^−2^ (Figure 3A–3D). These include four putative species of Actinostolidae, a family that includes the anemones *Pacmanactis* and *Marianactis* found on deep-sea hydrothermal vents elsewhere. There is also a red anemone that is similar in appearance to *Chondrophellia* sp. or *Hormathia spinosa* (personal communication, E. Rodriguez, Division of Invertebrate Zoology, American Museum of Natural History). The most obvious gastropod is an undescribed peltospiroid species (Figures 3B, 3D, and 4), generally found in dense aggregations up to ∼1,000 m^−2^. A second common gastropod is a limpet of the genus *Lepetodrilus* (Figure 3D). Phylogenetic analysis of the mitochondrial cytochrome oxidase I gene of this limpet (GenBank accession number JN628254) and a range of other *Lepetodrilus* species, using Bayesian inference, places the ESR limpet as a sister taxon to *L. atlanticus* (Figure S2), with a sequence divergence from this species of 5.48%. This level of genetic divergence is consistent with that found between *Lepetodrilus* species within complexes of sister taxa where interspecific distances of between 3% and 15% have been observed [33]. This new species is ubiquitous in low-temperature diffuse flow, being found on bare rock, sulphides, *Kiwa* n. sp., peltospiroid gastropods, and stalked barnacles. On the carapace of *Kiwa* n. sp., a halo of pale colouration surrounding the limpets indicates where *Lepetodrilus* n. sp. is grazing epizoic microbes. *Lepetodrilus* species have also been found previously on the carapaces of bythograeid crabs [33], as well as on the shells of vent molluscs and the tubes of siboglinid worms [34]. Not as visually apparent, but abundant in sediment residue in the ROV sample bioboxes from vent and diffuse flow areas at E2 and E9, is a small species of provannid gastropod. Several unidentified octopi were also observed within hydrothermal vent fields at E9 (Figure 3F). The vent fauna also includes dense aggregations of a stalked barnacle morphologically consistent with the genus *Vulcanolepas* (Figures 2E, 3A, 3B, and 3E). Phylogenetic analyses using Bayesian inference of the histone H3 and 28S rDNA [35] of the ESR *Vulcanolepas* (GenBank accession numbers JN628252, JN628523) and stalked barnacles from other hydrothermal vents (Figure S3) confirmed that the ESR barnacles were most closely related to, but a distinct species from, *V. osheai* (divergence of 0.34% and 0.22%, respectively). The latter species was described from the Brothers Caldera, Kermadec Ridge, South West Pacific [36]. The ESR *Vulcanolepas* occurs at densities of up to ∼750 m^−2^, particularly at E9 along the broken edge of sheet lava bathed in diffuse vent flow, as well as forming erect, dense colonies on chimneys emitting diffuse flow. Also scattered throughout the vent systems at E2 and E9 are at least three species of the vent pycnogonid *Sericosura* (Figure 3D), with the larger species *Colossendeis* cf. *concedis* and *C.* cf. *elephantis* occurring on the peripheral areas of the vents (personal communication, C. Arango, Queensland Museum South Bank). As at other vent sites in the Pacific and Atlantic Oceans [37]–[39], swarms of an unidentified amphipod were observed at E9. Although a variety of echinoderms were found during our observations, only one species, a seven-armed sea star from the family Stichasteridae (personal communication, C. Mah, Smithsonian National Museum of Natural History), appeared to be vent endemic (Figure 3E). This undescribed species was indicative of the proximity of vents in our 2009 observations, and during the 2010 campaign was found both peripherally and in areas of low-temperature diffuse venting. We observed it feeding on vent fauna, especially *Kiwa* n. sp. and barnacles. Fish were generally uncommon at the vent sites, and the only species that were observed were several species of macrourids on the vent periphery and a zoarcid, several specimens of which were recovered in baited traps at E9.

**Table 2:** Dominant fauna at East Scotia Ridge vents E2 and E9.

Higher Taxon Levels			Species (or Lowest Taxonomic Identification)	
Phylum	Subphylum or Class	Taxon Level 3	E2	E9
**Porifera**	Demospongiae	Cladorhizidae	*Cladorhiza* n. sp. 1	*Cladorhiza* n. sp. 1
				*Abyssocladia* n. sp. 1
**Cnidaria**	Anthozoa	Hormathiidae		*Chondrophellia* sp. or *Hormathia spinosa*
		Actinostolidae		Actinostolidae n. sp. 1
			Actinostolidae n. sp. 2	Actinostolidae n. sp. 2
			Actinostolidae n. sp. 3	Actinostolidae n. sp. 3
			Actinostolidae n. sp. 4	
**Annelida**	Polychaeta		Polynoidae sp. 1	
				Polynoidae sp. 2
				Polynoidae sp. 3
				Polynoidae sp. 4
**Mollusca**	Gastropoda		Peltospiroidea n. sp.	Peltospiroidea n. sp.
			cf. *Protolira* sp.	cf. *Protolira* sp.
			*Lepetodrilus* n. sp.1	*Lepetodrilus* n. sp.1
			Provannid sp. 1	Provannid sp. 1
				Provannid sp. 2
	Cephalopoda			Octopodidae
**Arthropoda**	Crustacea	Cirripedia	*Vulcanolepas* n. sp.	*Vulcanolepas* n. sp.
		Anomura	*Kiwa* n. sp.	*Kiwa* n. sp.
	Pycnogonida		*Sericosura* sp. 1	*Sericosura* sp. 1
			*Sericosura* sp. 2	*Sericosura* sp. 2
				*Sericosura* sp. 3
			*Colossendeis* cf. *concedis* (vent periphery)	*Colossendeis* cf. *concedis* (vent periphery)
			*Colossendeis* cf. *elephantis* (vent periphery)	*Colossendeis* cf. *elephantis* (vent periphery)
**Echinodermata**	Asteroidea	Stichasteridae	Stichasteridae n. sp.	Stichasteridae n. sp.
		Freyellidae	*Freyella* cf. *fragilissima*	*Freyella* cf. *fragilissima*
**Chordata**	Vertebrata			Zoarcid fish

All identifications are putative and await detailed taxonomic and molecular analysis.

**Figure 5:**
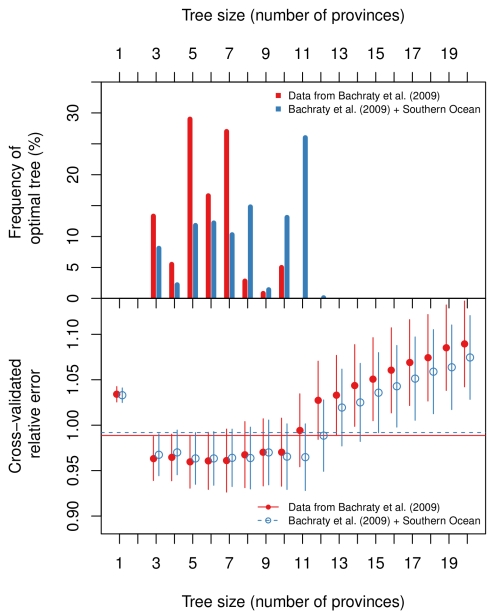
Selection of the multivariate regression tree for the global datasets of vent species. The datasets are species data from Bachraty et al. [8] (red/filled circles/solid line) and the same dataset with Southern Ocean vent sites added (blue/open circles/dashed line). Top panel: Frequency plot of the optimal tree size for 1,000 multiple cross-validations. The most common optimal tree size was five or seven provinces for the Bachraty et al. [8] dataset and 11 provinces for the combined dataset. Bottom panel: The cross-validated relative error indicates that predictive power is similar for a wide range of tree sizes. Vertical bars indicate ± one standard error, and the horizontal lines indicate one standard error above the minimum cross-validated relative error.

**Figure 6:**
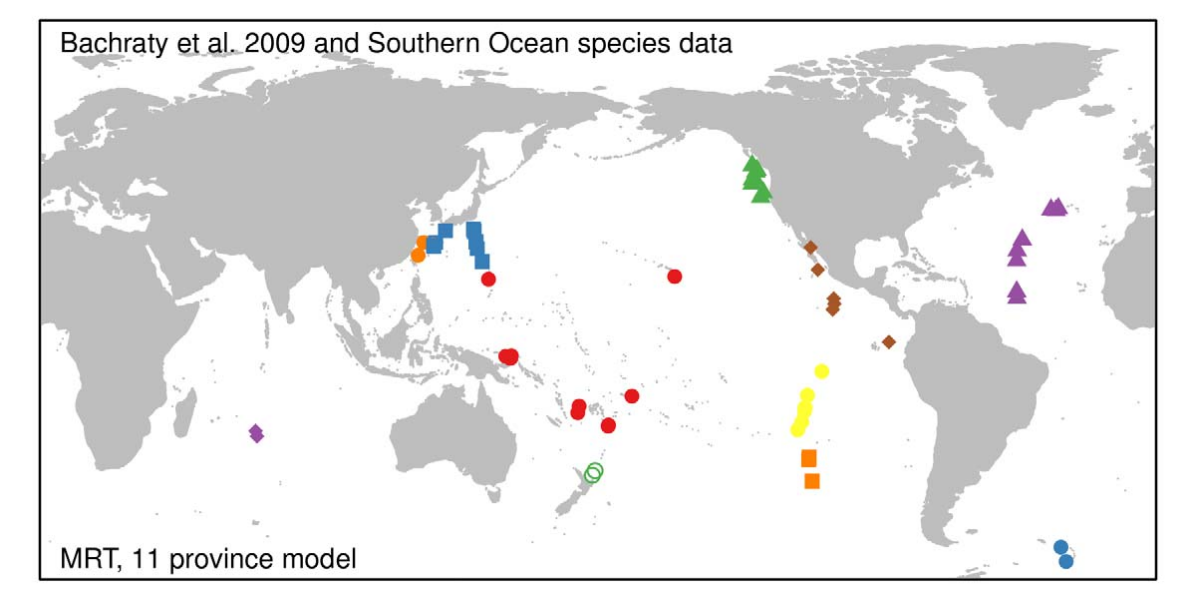
Results of geographically constrained clustering using multivariate regression trees. An 11-province model based on the combined dataset was the most frequent optimal model when using multiple cross-validations. Vent provinces are resolved comprising the Mid-Atlantic Ridge, the ESR, the northern, central, and southern East Pacific Rise, a further province located south of the Easter Microplate, four provinces in the western Pacific, and a further Indian Ocean province.

In order to examine how the fauna at E2 and E9 fit into the current understanding of the biogeography of deep-sea hydrothermal vents, we undertook an analysis of the global dataset on species presence/absence of most of the known hydrothermal vent communities using multivariate regression trees (MRT) after Bachraty et al. [8], but with modifications (see Materials and Methods). The MRT analyses, with cross-validation, produced a series of trees, many of which were only marginally worse than the best predictive tree (Figure 5). The optimal tree size, based on cross-validation error, varied between three and ten provinces for the Bachraty et al. [8] dataset and three and 11 provinces for the Bachraty et al. [8] dataset plus E2 and E9 (combined dataset). The most common optimal trees were the five- and seven-province models for the Bachraty et al. [8] dataset (Text S1; Figure S4A) and an 11-province model for the combined dataset (Figure 6). The six-province model proposed by Bachraty et al. [8] was not found to be the most frequently selected optimal tree. The 11-province model retained the Atlantic and East Pacific clusters but split up the Indo-Pacific province into five smaller clusters (Figure 6). In all iterations of the model (Figures 6 and S4) the sites south of the Easter Microplate in the South Pacific formed a separate cluster from all other East Pacific sites. E2 and E9 form a separate cluster for the optimal 11-province model (and seven-province model; see Figure S4) for the combined dataset, also suggesting that these sites form a new biogeographic province (but see discussion on the MRT method).

## Discussion

### Implications for Antarctic Biodiversity

Recent investigations of the deep-sea ecosystems of the Southern Ocean have revealed a high proportion of previously undescribed species, many of which are unknown from elsewhere [40]. Particularly notable in this respect are groups of the Isopoda, Ostracoda, Gastropoda, and Nematoda. It has been suggested that Southern Ocean species of these groups are not found outside of the Southern Ocean because they have life histories that are characterised by a low potential for dispersal [40]. Likewise, analyses of the fauna of the shelf and slopes of the islands of the Scotia Arc, as far north as Shag Rocks, suggest that the fauna is largely composed of Antarctic endemics [41]. The finding of a unique vent-endemic fauna within the Southern Ocean is consistent with this pattern of species distribution and is further evidence of the high regional endemism of the Antarctic marine biota. This study also provides the first identification and description, to our knowledge, of high-biomass hydrothermal-vent-endemic chemosynthetic communities in the Southern Ocean. Exploration of deep-sea hydrothermal vents in other sectors of the Southern Ocean, such as the Pacific-Antarctic Ridge [16], are likely to reveal further chemosynthetic communities.

### Implications for Hydrothermal Vent Biogeography

The fauna observed at the vents along the ESR contains none of the dominant vent species normally found at vents along the main mid-ocean ridge systems. The ESR sites are notable for the absence of siboglinid tubeworms, alvinellid polychaetes, vesicomyid clams, bathymodiolid mussels, and alvinocaridid shrimp. In addition, there is an absence of typical predators such as bythograeid crabs. Species found at the ESR vents include anemones, lepetodrilid limpets, provannid gastropods, stalked barnacles, and at least three species of pycnogonids, thus these vents share some faunal elements with communities found at vents associated with back-arc basins in the West and South West Pacific, the mid-ocean ridge in the South East Pacific, and the Mid-Atlantic Ridge. The dominant species at the ESR vents is an anomuran crab of the genus *Kiwa*, which has congeneric species along the Pacific-Antarctic Ridge and at cold seeps off Costa Rica [9],[42].

Connections among the biogeographic provinces identified over the last ten years are consistent with dispersal of taxa along mid-ocean ridge systems, with vicariance events being related to severance of ridges through subduction or other processes [43]. This connectivity is also consistent with gene-flow studies that have demonstrated significant relationships between measures of genetic differentiation (*F*_ST_) and whether populations are present on the same ridge segment, are separated by transform faults, or are present on different ridges [6],[44]. However, the biogeographic patterns exhibited by hydrothermal vent communities may also be influenced by larval dispersal on deep-ocean currents that do not follow the line of ridge axes, with or without the aid of evolutionary stepping stones provided by other chemosynthetic ecosystems such as cold seeps and whale falls [6]–[8]. Examples of where such dispersal routes may have been important include the dispersal routes between the eastern Pacific and Mid-Atlantic Ridge, and the eastern Pacific, South Atlantic, and Indian Ocean [7],[8].

Our data from vents at E2 and E9 along the ESR provide three lines of evidence that the fauna at these sites represents a separate and new biogeographic province from those previously described for the global ocean [7],[8]. First, the taxa of the vent fields at E2 and E9 are distinct from those of other provinces at least at the species level (e.g., *Kiwa* n. sp., *Vulcanolepas* n. sp., and *Lepetodrilus* n. sp.). Second, the structure of the assemblages differs from that of other provinces where fauna are shared at higher taxonomic levels. For example, at the nearest vent site where another species of *Kiwa* has been reported (*K. hirsuta*; 38°S, Pacific-Antarctic Ridge), that species occurs in the periphery with a reported population density of 0.1–0.2 m^−2^, and in diffuse venting areas along with other widespread vent fauna, such as *Bathymodiolus* sp. and bythograeid crabs [9]. In contrast, at the ESR vents, *Kiwa* n. sp. occurs at high population densities (∼600 m^−2^) proximal to fluid exits, in the niches usually taken by taxa such as alvinellid polychaetes [45] or aggregations of alvinocaridid shrimp [46]. Also distinct in the assemblages of the ESR vents is the variety of vent-endemic anemones, and the presence of an undescribed seven-arm stichasterid sea star as a predator, and a conspicuous rarity of polychaetes, other than polynoid scale worms. Finally, the MRT analyses of the combined dataset indicate that using the most common optimal tree, E2 and E9 form a separate cluster from other vent provinces. These analyses also indicated that several other areas, especially the eastern Pacific vent sites south of the Easter Microplate, consistently form a separate biogeographic province in a range of optimal trees. This region has been recognised as a biogeographic boundary, known as the Easter Microplate boundary, in several other studies [6].

With regards to the third line of evidence, the MRT results should be interpreted with care. First, the Indian Ocean, South East Pacific Rise, and Antarctic sites are significantly undersampled compared to sites in the northern and central East Pacific Rise, the Mid-Atlantic Ridge, and western Pacific back-arc basins. Second, the species lists presented in Bachraty et al. [8] do not account for many of the cryptic species that have been identified amongst some groups of vent taxa (e.g., *Lepetodrilus* [33]). Both of these factors introduce significant potential errors into the resolution of biogeographic patterns of the vent fauna using multivariate methods. Notwithstanding these problems, our analysis failed to reproduce the six-province model proposed by Bachraty et al. [8], and we see two major problems with their analysis. The first concerns the stability of the statistical method they used; the second concerns the choice of constraining variables for the cluster analysis. Regarding stability, the MRT method does not give a clear preference to a certain number of provinces, but rather a series of similarly “good” trees. The reason for the choice of the six-province model, given the data of Bachraty et al. [8], is unclear. Breiman et al. [47] recommend picking the smallest tree within one standard error of the minimum tree when there is no clear optimum, which would lead to a model with three provinces for both the Bachraty et al. [8] dataset and the combined dataset used in this study. We chose instead to present models with more than three provinces in this study, based on the results of multiple cross-validation. However, we suspect that the lack of stability of tree size is based on a combination of two things. First, vent biogeographic provinces appear to be hard to resolve based on the current presence/absence data alone. This idea is supported by the marginal differences between a range of preferential trees in the MRT (Figure 5) and by variation in the results across a number of unconstrained agglomerative cluster analyses we undertook whilst exploring the Bachraty et al. [8] and combined datasets for this study (see Text S1 and Figure S5). It is also notable that studies of other deep-sea ecosystems have demonstrated that analyses of species presence/absence can miss significant differences in the structure of marine communities that can be resolved using species abundance or ranked abundance data (e.g., seamounts [48]). Secondly, we think that latitude and longitude are not a sensible choice of constraining variables both from a mathematical and a biological perspective. In the MRT analysis, latitude and longitude are effectively treated as Cartesian coordinates, which is not an appropriate representation of geographic distances on the Earth's surface. This introduces a bias where sites at high latitude appear to be more distant along a latitude circle than sites at low latitude. Furthermore, by encoding the longitude into 0–360° east of Greenwich, Bachraty et al. [8] introduce the implicit assumption that the Atlantic and Indo-Pacific are two extremes of the spatial spectrum, whereas in reality the two are joined around the Cape of Good Hope. Not surprisingly, different representations of longitude yield different MRT results, both in terms of optimal tree size and in the assignment of sites to “provinces” (see Text S1 and Figure S6). Apart from this geographical issue, present-day locations may not be good predictors for vent biogeography as they neither reflect geographic proximity on evolutionary time scales nor take into account other features and processes that are thought to influence deep-sea biogeography. These include factors such as depth, topography, currents, and oceanic fronts, many of which can act as variable dispersal filters [6],[49]–[51].

Overall, our evidence for a separate biogeographic province for the ESR is consistent with the history and present physical environment of the Southern Ocean. The Southern Ocean is separated from the remaining global ocean by the surface-to-seabed Polar Front [52], which is a major barrier to dispersal of fauna to and from Antarctic waters [53]. This region represents a sharp boundary in physical conditions that was established after the initiation of the Antarctic Circumpolar Current and became more extreme at the middle Miocene climate transition (∼13.8 Mya [54]), a time that is close to the initiation of spreading at the ESR. Taxa commonly found in the rest of the world's oceans, such as brachyuran crabs and decapod lobsters, are absent from the Antarctic, and the non-vent marine fauna of the Southern Ocean is highly endemic [40],[55],[56]. Explanations for this have included physiological barriers, an example being the decapod crustaceans, which have an inability to down-regulate blood magnesium levels sufficiently below that of seawater, leading to a loss of activity and eventual death at polar water temperatures [57]. It is also notable that a high proportion of Antarctic marine invertebrates have life histories that include direct or lecithotrophic larval development, although some common species, associated with unstable habitats, exhibit planktotrophy [58]. The reason for this is uncertain, although it is likely to be an adaptation to the extreme seasonality of the Antarctic and poor food supply for large parts of the year [58]. With the exception of the Siboglinidae, the taxa that are absent from the vents of the ESR have planktotrophic larval development (including the alvinocaridid shrimp and vent mussels). It is notable that the deep-sea vent ecosystems recently described from the Arctic also show an absence of vent shrimp and vent mussels [12]. In the Arctic, the niche usually occupied by shrimp in Atlantic vent fields is occupied by an amphipod with chemoautotrophic gill symbionts [12]. The biological filter represented by the Polar Front may thus explain the absence of bythograeid crabs, shrimps of the Alvinocarididae, and other taxa commonly associated with vents.

Current flow south of the Polar Frontal Zone is dominated by the eastward-flowing ACC, and maps of potential vorticity and evidence from ocean tracers of the high southern latitudes give rise to the possibility of larva-mediated dispersal and faunal similarities among the disjunct South East Pacific Rise, Chile Rise, ESR, and southernmost Mid-Atlantic Ridge [7],[59]. Our observations are consistent with this hypothesis in the identification of a new species of *Kiwa*, other species of which has been found on the Pacific-Antarctic Ridge and on the continental slope of Costa Rica. Other faunal elements may also be shared between the vents on the ESR and those of the South East and South West Pacific. Early investigation of the life history of *Kiwa* n. sp. from the ESR also suggests that the larvae are brooded and hatch from eggs at a morphologically advanced stage, which is probably not conducive to long-distance dispersal in deep water. However, inferring dispersal capability from life history characteristics should be undertaken with caution, given that life history only partially explains the observed patterns of gene flow for other marine species [60],[61]. A test of the importance of current-mediated dispersal in the evolution of communities at the ESR would be faunal and phylogenetic comparisons of this community with the biota present at the South East Pacific Rise and Chile Rise, along with that at vents along the Antarctic Peninsula in the Bransfield Strait [14].

The discovery of vent biota on the ESR with faunal connections to other southern hemisphere vent systems, including those in both the Pacific and the Atlantic, suggests a more complex picture of vent biogeography than previously considered. A full understanding of the relationships of the fauna of the ESR vents with those elsewhere will only be realised with complete analyses of the fauna collected at 56°S and 60°S at the ESR, and the location and documentation of further hydrothermal vent communities at high latitudes in the Southern Ocean and southern Pacific, Atlantic, and Indian Oceans. Further exploration of high-latitude ridges is critical for a full understanding of the global biogeography of vent ecosystems, given the potential role of the Southern Ocean as a gateway or a barrier between the major ocean ridges and back-arc basins.

Finally, our direct observations of hydrothermal vent fields south of 40°S latitude in the southern hemisphere represent the culmination of a 30-y poleward trend in hydrothermal exploration, which began at low latitudes. However, a seafloor image taken as long ago as 1966 at 2,377 m depth on ESR segment E9 shows a faunal assemblage similar to that which we now identify as associated with hydrothermal vents on this segment [62]. Thus, it appears that a vent community may have been observed but not recognised at high latitudes a decade prior to the original discovery of vent communities in the Galápagos Rift [1]. It is interesting to reflect that if this seafloor assemblage had been investigated in greater detail at that time, the entire history of global-scale hydrothermal exploration could have followed a quite different path.

## Materials and Methods

### Bathymetric and Geophysical Surveys

Two modes of geophysics data acquisition were carried out during the cruises: (1) ship-based geophysical survey and (2) ROV geophysical survey. On both RRS *James Clark Ross* 224 and RRS *James Cook* 042, ship-based geophysical data collection consisted of seafloor mapping using hull-mounted Kongsberg-Simrad EM120 multibeam echo sounders, and sub-bottom profiling using a hull-mounted parametric echo sounder. ROV geophysics data collection consisted of high-resolution seafloor mapping using the ROV *Isis* Simrad SM2000 multibeam echo sounder. Few ship-based surveys were carried out during the RRS *James Cook* 042 cruise.

### Water Column Sampling

The majority of the water samples were collected using a Seabird +911 CTD on a titanium frame with up to 24 externally sprung Niskin bottles. This is a clean system, specifically designed for the sampling of waters with low levels of trace metals and nutrients. The bottles are Teflon lined, with Teflon taps and non-metallic parts; any metallic components are titanium or high-quality stainless steel.

The CTD Carousel Niskin and ROV Mini-Niskin bottles were sampled for (in order): (1) methane (125 ml, poisoned with HgCl for analysis at the National Oceanography Centre, Southampton [NOC]); (2) dissolved inorganic carbon (250 ml, poisoned with HgCl for analysis at NOC); (3) total dissolved organic carbon (20 ml, filtered through a 0.2-µm filter and acidified with HCl for analysis at NOC); (4) trace metals (filtered through a 0.2-µm filter into an 500-ml acid-cleaned LDPE bottle for analysis at NOC); (5) metal speciation (filtered through a 0.2-µm filter into duplicate 250-ml bottles and frozen for analysis at NOC); and (6) siderophores—remaining volume for Mini-Niskin bottles, usually 10 l for large Niskin bottles—filtered and sucked through an Isolute ENV+ column (frozen) for characterisation at NOC. Finally, the filters were all washed for salts with Milli-Q water (pH 8) and stored frozen for analysis at NOC.

### Hydrothermal Fluid Sampling

Collection of these samples was achieved using titanium (Ti) samplers, equipped with an inductively coupled link (ICL) high-temperature sensor to ensure the collection of high-quality samples. In the case of diffuse flow, or for sampling of friable chimney structures, the Ti samplers were used in conjunction with a specially constructed Ti diffuse sampler, which was used to prevent entrainment of surrounding seawater into the path of the fluid during sampling.

The Ti samplers were cleaned thoroughly before deployment using a solvent flux remover and rinsed with Milli-Q water. All Ti–Ti surfaces were lubricated with Fluorolube. Sample bottles were deployed in pairs, although each bottle had its own nozzle for insertion into the vent orifice (or diffuse flow sampler). Each pair of Ti samplers was coupled to an ICL high-temperature sensor that was located at the tip of the sample nozzles. Pins for firing the bottles were set at a distance of 22–31 mm above the top of the Ti sampler; however, when the pins were set high (31 mm), it proved difficult to couple the ICL temperature probe (for this reason, no temperature was recorded for some samples). The optimal setting for the pins was found to be ∼27 mm.

### Diffuse Flow Sampling

For optimal sampling of diffuse flow, the diffuse flow sampler was placed over the area to be sampled, and allowed to equilibrate until fluid was observed to be flowing out of the sampler. The nozzles of the Ti samplers were then inserted into the diffuse flow sampler, and the ram was slowly lowered until a reading was obtained on the ICL sensor. Once the temperature reading was considered to be steady, sampling proceeded in the same way as for a focussed fluid.

As soon as the samplers returned to the surface, they were rinsed in Milli-Q water, and the fluid was withdrawn. Separate sub-samples were collected for (1) refractive index, (2) alkalinity, (3) dissolved inorganic carbon and carbon isotopes, (4) pH, (5) gases (including CH_4_, CO_2_, and H_2_), (6) anions and silica, (7) nutrients, (8) dissolved organic carbon, (9) O and H isotopes, and (10) bacteria, in that order. The remainder of the sample was emptied into an acid-cleaned 1-l HDPE bottle for analysis of all other constituents, including cations and the transition metals. Any residue remaining in the bottle was washed in to an acid-clean 30-ml HDPE bottle with Milli-Q water.

Analysis of “time-critical” parameters (e.g., pH) and key indicators of sample quality (e.g., Cl) was carried out onboard. Other constituents were transported back to NOC for analysis over the following 18 mo.

### Microbiology

Samples were taken on the RRS *James Clark Ross* 224 cruise (January–February 2009). Water from within the buoyant vent plume was sampled with a CTD. Two litres of water was filtered through a 0.2-µm pore-size nitrocellulose filter. The filters were frozen at −80°C until further analysis. DNA was extracted from the filters using a phenol/chloroform protocol [63]. The 16S rDNA gene was amplified by PCR using the universal primers 27F and 1492R. PCR conditions were 3 min at 94°C, followed by 30 cycles of 60 s at 94°C, 45 s 50°C, 90 s at 72°C, and a final elongation of 5 min at 72°C. PCR products were cloned into the pCR2.1 vector by TOPO TA cloning (Invitrogen), following the manufacturer's recommendations and plated on LB-ampicillin plates containing X-gal for blue-white screening. White clones were checked for correct insert size by PCR using the plasmid primers M13F and M13R. In total, 285 clones (166 from E2 and 119 from E9) were sequenced from the 3′ end by Sanger sequencing at LGC Genomics. The average sequence length was 885 bp. The sequences were trimmed and quality-control checked with the software package Geneious [64] and subsequently aligned to a reference database (SILVA, version 102 [65]) and identified phylogenetically within ARB [66].

### Faunal Imaging

Two equipment arrangements were used to conduct video-graphic surveys during ROV *Isis* dives. “Horizontal” surveys (surveys of horizontal substratum) were undertaken using a downward-looking Atlas three-chip charge coupled device video camera. The camera housing was mounted to view the seafloor through an aperture cut in the port forward corner of the ROV tool tray. A downward-facing HMI light was similarly mounted through the starboard forward corner of the tool tray. Two parallel lasers, 0.1 m apart, were mounted parallel to the focal axis of the camera to provide scale in images. Footage from the downward-looking Atlas camera was recorded to DVCAM tapes and DVD in the ROV control van. Controls for the Atlas camera (iris, zoom, focus, and colour balance) were adjusted from the ROV control van to obtain the clearest possible images for faunal identification.

“Vertical” video-graphic surveys (surveys of vertical substrata such as vent chimneys) were undertaken using the high-definition pilot pan-and-tilt camera of the ROV *Isis*. For these surveys, this camera was configured to view horizontally forwards from the vehicle, so that its focal axis was perpendicular to vertical substratum surfaces. Two parallel lasers, 0.1 m apart, were mounted parallel to the focal axis of the camera to provide scale in images.

Vertical surveys were undertaken using closed control of the ROV to maintain constant vehicle heading, and Doppler lock to enable movements of the vehicle over precise distances relative to the seafloor. These features enabled the ROV to undertake vertical lines up and down chimneys, offset by fixed horizontal distances, to obtain overlapping video images of the structure from a particular heading. Distance from the vehicle to the structure was kept constant, so that survey lines lay on a flat vertical plane a fixed distance from the structure being surveyed. Camera zoom was set in vertical surveys to achieve image frames approximately 1 m wide, with no adjustments during lines, and images were subsequently mosaicked together from overlapping lines for analysis.

Faunal samples were collected either by suction sampler or by scoop and brought to the surface in ambient seawater. Once on board, samples were immediately transferred to cold water in the controlled temperature laboratory (∼4°C), where individuals were dissected and either frozen or stored in molecular grade ethanol for molecular analysis, frozen for isotope analysis, or fixed in 10% seawater formalin for morphological analysis.

### Molecular Studies

#### DNA isolation, amplification, and sequencing

Genomic DNA was isolated from tissue samples of selected specimens, using different tissues depending on the taxon (*Kiwa* n. sp./pycnogonid: muscle tissue in merus/femur; gastropod/anemone: foot). DNA was extracted with the DNeasy Tissue Extraction Kit (Qiagen) as directed by the manufacturer.

Targeted gene regions were amplified via PCR using one or more sets of primers. Reactions were performed in 25-µl volumes, containing 2 µl of each primer (forward and reverse) at a concentration of 10 pmol/µl, 16 µl of Qiagen HotStarTaq Master Mix, 4 µl of DNA template (∼50 ng), and 1 µl of double-distilled water. Cytochrome oxidase I reactions were performed in 10-µl volumes, containing 0.5 µl of each primer (forward and reverse) at a concentration of 10 nmol, 5 µl of Qiagen 10× PCR buffer, 1.5 µl of MgCl_2_ (25 mM), 1 µl of dNTPs (2 nmol, Bioline), 0.25 µl of Taq (5 U/µl), and 1 µl of DNA template (∼30 ng).

Primers were selected from existing papers or, if no amplification or poor amplification occurred, were designed on the basis of existing sequence data or initial sequence data obtained in this study.

#### PCR primers

For *Kiwa* n. sp., one mitochondrial gene (16S) and two nuclear genes (18S and 28S) were selected (Table S1), as these genes had previously been shown to be good markers for resolving relationships across broad time scales in the Crustacea [67]. For *Vulcanolepas*, 28S rDNA and the histone gene H3 were selected (Table S2), owing to their use in previous barnacle phylogenetic reconstructions [35],[68]. For *Lepetodrilus*, the mitochondrial cytochrome oxidase I was amplified using LCO 1490 and HCO 2198 [69] (Table S3), following the use of this gene to resolve the molecular phylogeny of the Vetigastropoda [70]. PCR cycling protocols were as follows. For *Kiwa* n. sp. 16S rDNA: initial HotStarTaq denaturation at 95°C for 15 min, followed by 40 cycles of 94°C for 45 s, 55°C for 90 s, 72°C for 1 min, and a final extension of 7 min at 72°C. For 18S rDNA: initial HotStarTaq denaturation at 95°C for 15 min, followed by 30 cycles of 94°C for 1 min, 50°C for 90 s, 72°C for 2 min, and a final extension of 7 min at 72°C. For 28S rDNA: initial HotStarTaq denaturation at 95°C for 15 min, followed by 30 cycles of 94°C for 1 min, 55°C for 90 s, 72°C for 2 min, and a final extension of 7 min at 72°C. For *Vulcanolepas* histone gene H3: initial HotStarTaq denaturation at 95°C for 15 min, followed by 50 cycles of 95°C for 1 min, 50°C for 1 min, 72°C for 1 min, and a final extension of 5 min at 72°C. For 28S rDNA: initial HotStarTaq denaturation at 95°C for 15 min, followed by 35 cycles of 94°C for 1 min, 55°C for 1 min, 72°C for 90 s, and a final extension of 7 min at 72°C. For *Lepetodrilus* cytochrome oxidase I: initial HotStarTaq denaturation at 94°C for 15 min, followed by five cycles of 94°C for 1 min, 45°C for 1.5 min, 72°C for 1.5 min, then 30 cycles of 94°C for 1 min, 50°C for 1 min, 72°C for 1 min, and a final extension of 5 min at 72°C.

All PCR reactions and some sequencing reactions were performed on a Bio-Rad C1000 Thermal Cycler. PCR product was purified using QIAquick PCR Purification Kit (catalog number 28106). Where the C1000 Thermal Cycler was used for sequencing reactions, an Applied Biosystems 3100 DNA Analyser was used for sequencing. In all other cases, PCR product was sent to the Macrogen Europe Laboratory, where sequencing was conducted under BigDye terminator cycling conditions; the reacted products were purified using ethanol precipitation and run using an ABI 3730XL Automatic Sequencer. Forward and reverse sequences were assembled and cleaned using the computer program Sequencher 3.0.

#### Molecular data analyses

Alignments were carried out using MUSCLE [71],[72]. For ribosomal genes, gaps were treated as informative events and were added as characters to the end of the sequences using the software FastGap 1.2 [73]. All quoted genetic distances are Tamura-Nei distances calculated using the software MEGA 4.1 [74]. Simple p-distances will tend to underestimate true genetic distances owing to the possibility of multiple nucleotide substitutions (“hits”) at the same locus. The Tamura-Nei model corrects for multiple hits, whilst taking into account the differences in substitution rate and nucleotide frequencies.

#### Divergence dates for *Kiwa* n. sp. versus *K. hirsuta*

There are no substitution rate estimates for anomuran crustaceans for 18S and 28S. For 16S, there is an estimate for porcelain crabs based on divergence of two populations of a species (*Petrolisthes armatus*) isolated from each other by the formation of the Panama Isthmus 3.5 Mya. Stillman and Reeb [31] estimate a divergence rate of 0.53% per million years. The ESR crab has a 6.45% difference to *K. hirsuta*, which would place a divergence date at 12.2 Mya.

There are other substitution rate estimates for crustaceans, such as 0.65% per million years for Jamaican crabs [75], 0.9% per million years for fiddler crabs [76], and 0.67% per million years for a group of North American barnacles [77]. These substitution rates are calculated by dividing the percentage difference of presumed sister or cryptic species from either side of the Panama Isthmus by the estimated date that the Isthmus was formed (3.5 Mya). The 0.53% divergence rate estimate [31] is the preferred estimate as it is the only one for anomurans. Furthermore, it is likely that many populations became isolated from each other before the final closing of the Panama Isthmus, and therefore substitution rate estimates are likely to have been overestimated rather than underestimated, and more conservative rates are likely to be a better reflection of evolution. For these reasons, the tentative 12.2-million-year divergence date calculated for the ESR crab and *K. hirsuta* is likely to be more recent than the real date of divergence. It should be noted also that the 0.53% divergence rate estimated by Stillman and Reeb [31] is based on a tropical shallow-water species of anomuran, and substitution rates for deep-sea crustaceans may be very different.

#### Phylogenetic analyses

Phylogenetic trees were generated for *Kiwa* n. sp., *Vulcanolepas* n. sp., and *Lepetodrilus* n. sp. in order to reveal their affinity to other vent fauna. Alignments with sequences obtained in this study and sequences from GenBank were constructed using MUSCLE with the software MEGA 4.1 [73]. For ribosomal genes, gaps were treated as informative events and were added as characters to the end of the sequences using the software FastGap 1.2 [74]. Bayesian inference of phylogeny was performed using MrBayes 3.1.2 [78]. Appropriate substitution models for different genes were determined using jModelTest 0.1.1 [79] using the Akaike Information Criterion (AIC). For each of the three species investigated, Metropolis coupled Monte Carlo Markov Chains were run for 5 million generations in two simultaneous runs, each with four differently heated chains. Topologies were sampled every 100 generations, and the first 12,500 trees (25%) were discarded as “burn in”.

#### Species-specific methods

For *Kiwa* n. sp., a 414-bp fragment of the mitochondrial ribosomal gene 16S was used for the phylogenetic analysis (see Table S4). With gaps coded in the final alignment, the length was 495 bp. The substitution model with the best AIC score was the generalised time reversible model with a gamma distribution. For *Vulcanolepas* n. sp., a 296-bp fragment of nuclear protein-coding gene H3 and a 903-bp fragment of the nuclear ribosomal gene 28S were used for the phylogenetic analyses (see Table S5). Gaps in the final 28S alignment were coded for, and the two separate alignments were concatenated to create a final alignment 1,223 bp long. In the Bayesian analysis using MrBayes 3.1.2, the concatenated dataset was partitioned into the two gene regions, as the substitution models used were different. Based on AIC scores in jModelTest, the Hasegawa, Kishino, and Yano model with gamma distribution and invariable sites was used for the H3 fragment and the Felsenstein 1981 (F81) model was used for the 28S fragment. For *Lepetodrilus* n. sp., a 522-bp fragment of the mitochondrial protein-coding CO1 gene was used for the phylogenetic analysis (see Table S6). Based on AIC scores in jModelTest, the Hasegawa, Kishino, and Yano model with gamma distribution and invariable sites was used in the Bayesian analysis.

### Multivariate Analysis

Geographically constrained clustering was performed to investigate the biogeographic placement of the Southern Ocean hydrothermal vents in the global classification scheme proposed by Bachraty et al. [8] using MRT [80]. For this analysis, data were subjected to a Hellinger transformation [81]. Trees were then computed using the “mvpart” package [82] in the *R* environment for statistical computing [83]. Optimal tree size was investigated by running 1,000 multiple cross-validations on each dataset.

## Supporting Information

Figure S1 **Phylogenetic tree for Anomura based on 16S rDNA.** Phylogenetic tree showing the relationships of anomurans, including *Kiwa* n.sp., derived from a 495-base-pair sequence of the mitochondrial 16S rDNA gene based on Bayesian inference. Values above nodes are Bayesian posterior probability values. Scale bars indicate percent sequence divergence. All nodes with *p*<0.5 were collapsed into basal polytomies.
(TIF)

Figure S2 **Phylogenetic tree for** ***Lepetodrilus*** **based on cytochrome oxidase I.** Phylogenetic tree showing the relationships of limpets of the genus *Lepetodrilus*, including *Lepetodrilus* n. sp. from the ESR (*Pseudorimula* is used as the outgroup), derived from a 522-base-pair fragment of the mitochondrial cytochrome oxidase I gene based on Bayesian inference. Values above nodes are Bayesian posterior probability values. Scale bars indicate percent sequence divergence. All nodes with *p*<0.5 were collapsed into basal polytomies. CIR, Central Indian Ridge.
(TIF)

Figure S3 **Phylogenetic tree for** ***Vulcanolepas*** **based on histone H3 and 28S rDNA.** Phylogenetic tree showing the relationships of stalked barnacles, including *Vulcanolepas* n. sp., derived from a concatenated sequence of histone H3 and nuclear 28S rDNA gene 1,223 base pairs in length based on Bayesian inference. Values above nodes are Bayesian posterior probability values. Scale bars indicate percent sequence divergence. All nodes with *p*<0.5 were collapsed into basal polytomies.
(TIF)

Figure S4 **Multivariate regression trees for seven province models using the Bachraty et al.** [**8**] **and combined datasets.** (A) Results of geographically constrained clustering using MRTs and a seven province model based on the data from Bachraty et al. [8]. This model recovers all provinces proposed by Bachraty et al. [8], with an additional split in the South East Pacific Rise. (B) Results of geographically constrained clustering using MRTs and a seven-province model based on the data from Bachraty et al. [8] and the Southern Ocean sites described in this study. This model does not recover the North West Pacific province proposed by Bachraty et al. [8]; instead, it supports the additional split in the South East Pacific Rise, as well as a separate province for the Southern Ocean sites.
(TIF)

Figure S5 **Results of hierarchical agglomerative cluster analysis of community composition data at species level.** The tree is based on the Raup-Crick similarity coefficient, a probabilistic measure for presence/absence data.
(TIF)

Figure S6 **Selection of the multivariate regression tree for a global dataset of vent species using different representations of longitude.** The dataset is the species data from Bachraty et al. [8], with Southern Ocean vent sites added (combined dataset). Longitude representations are −180° to +180°, centred on Greenwich (red/filled circles/solid line), 0° to 360° east of 60°W (blue/open circles/dashed line) and 0 to 360° east of Greenwich (green/open triangles/dotted line). (A) Frequency plot of the optimal tree sizes for 1,000 multiple cross-validations. The most common optimal tree size was five and six provinces for the traditional −180° to 180° representation of longitude, five provinces for eastings from 60°W, and 11 provinces for eastings from Greenwich. (B) The cross-validated relative error indicates that predictive power is similar for a wide range of tree sizes. Vertical bars indicate ± one standard error, and the horizontal lines indicate one standard error above the minimum cross-validated relative error. (C and D) Geographic representation of the effects of different longitude encodings. The world map is shifted accordingly to illustrate the edges introduced by using latitude and longitude like Cartesian coordinates. Note the differing provinces in the East Pacific. (C) A five-province model based on the traditional −180° to 180° representation of longitude. (D) A five-province model based on eastings from 60°W.
(TIF)

Table S1 **For** ***Kiwa*** **n. sp., primers used for amplification and sequencing of 16S mitochondrial rDNA and 18S and 28S nuclear rDNA genes.**
(DOC)

Table S2 **For** ***Vulcanolepas*** **n. sp., primers used for amplification and sequencing of histone H3 and 28S nuclear rDNA genes.**
(DOC)

Table S3 **For** ***Lepetodrilus*** **n. sp., primers used for amplification and sequencing of cytochrome oxidase I.**
(DOC)

Table S4 **Sequences used for phylogenetic analysis of 16S rDNA to show the relationship of** ***Kiwa*** **n. sp. with other anomuran taxa.**
(DOC)

Table S5 **Sequences used for phylogenetic analysis of H3 and 28S rDNA to show the relationship of** ***Vulcanolepas*** **n. sp. with other stalked barnacles from deep-sea hydrothermal vents.**
(DOC)

Table S6 **Sequences used for phylogenetic analaysis of cytochrome oxidase I to show the relationship of** ***Lepetodrilus*** **n. sp. with other lepetodrilid limpets from deep-sea hydrothermal vents.**
(DOC)

Text S1 **Supplementary information.**
(DOC)

## Abbreviations

AIC: Akaike Information Criterion
CTD: conductivity–temperature–depth
ESR: East Scotia Ridge
ICL: inductively coupled link
MRT: multivariate regression tree
Mya: million years ago
NOC: National Oceanography Centre, Southampton
ROV: remotely operated vehicle
SHRIMP: Seabed High Resolution Imaging Platform
