# Supplementary figures and images for: The Discovery of New Deep-Sea Hydrothermal Vent Communities in the Southern Ocean and Implications for Biogeography
Source: PLoS Biol. 2012 Jan 3;10(1):e1001234. doi: 10.1371/journal.pbio.1001234 (PMC3250512; doi:10.1371/journal.pbio.1001234)

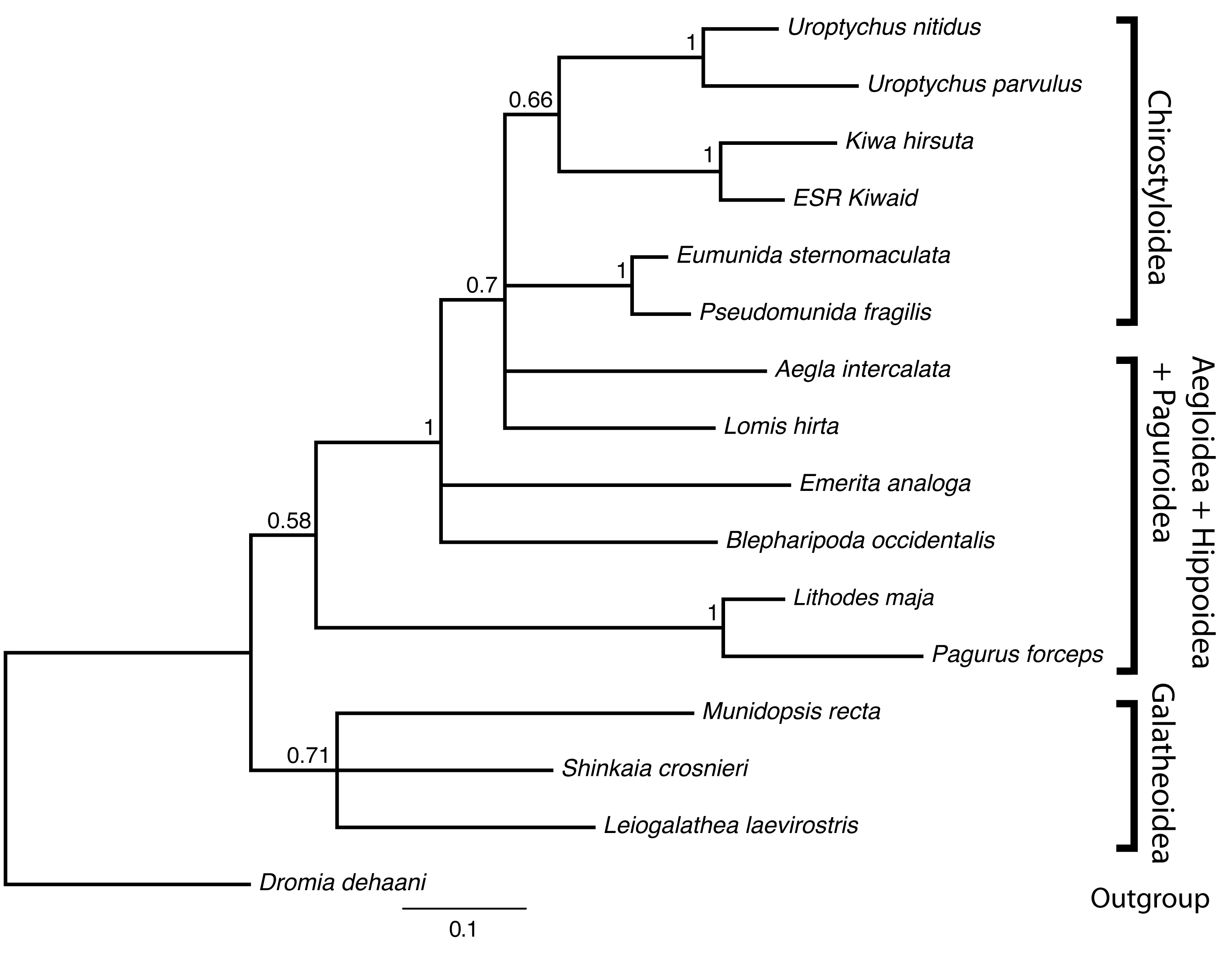

Supplement: Figure S1 — Phylogenetic tree for Anomura based on 16S rDNA. Phylogenetic tree showing the relationships of anomurans, including Kiwa n.sp., derived from a 495-base-pair sequence of the mitochondrial 16S rDNA gene based on Bayesian inference. Values above nodes are Bayesian posterior probability values. Scale bars indicate percent sequence divergence. All nodes with p<0.5 were collapsed into basal polytomies. (TIF) [file pbio.1001234.s001.tif]

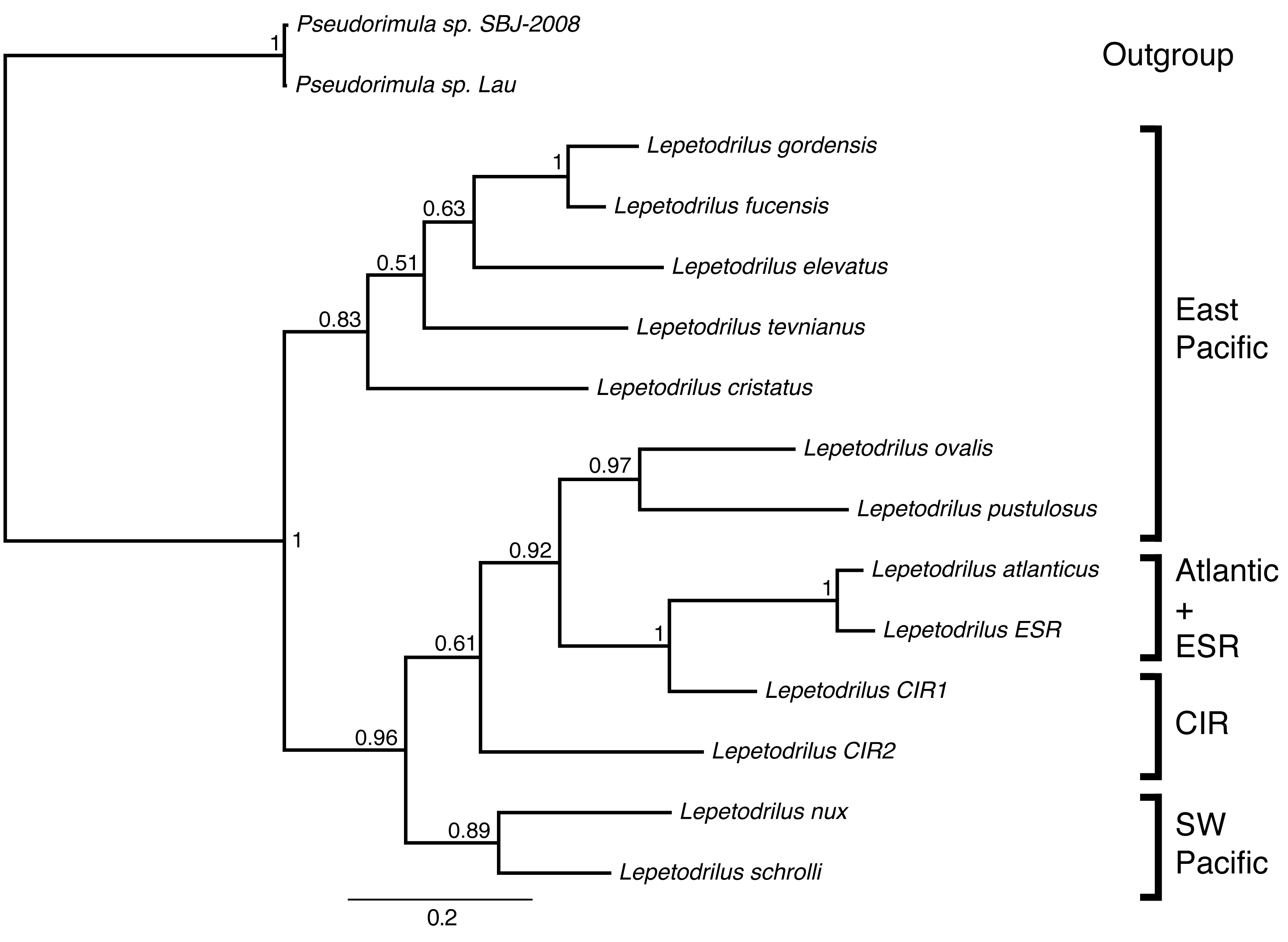

Supplement: Figure S2 — Phylogenetic tree for Lepetodrilus based on cytochrome oxidase I. Phylogenetic tree showing the relationships of limpets of the genus Lepetodrilus, including Lepetodrilus n. sp. from the ESR (Pseudorimula is used as the outgroup), derived from a 522-base-pair fragment of the mitochondrial cytochrome oxidase I gene based on Bayesian inference. Values above nodes are Bayesian posterior probability values. Scale bars indicate percent sequence divergence. All nodes with p<0.5 were collapsed into basal polytomies. CIR, Central Indian Ridge. (TIF) [file pbio.1001234.s002.tif]

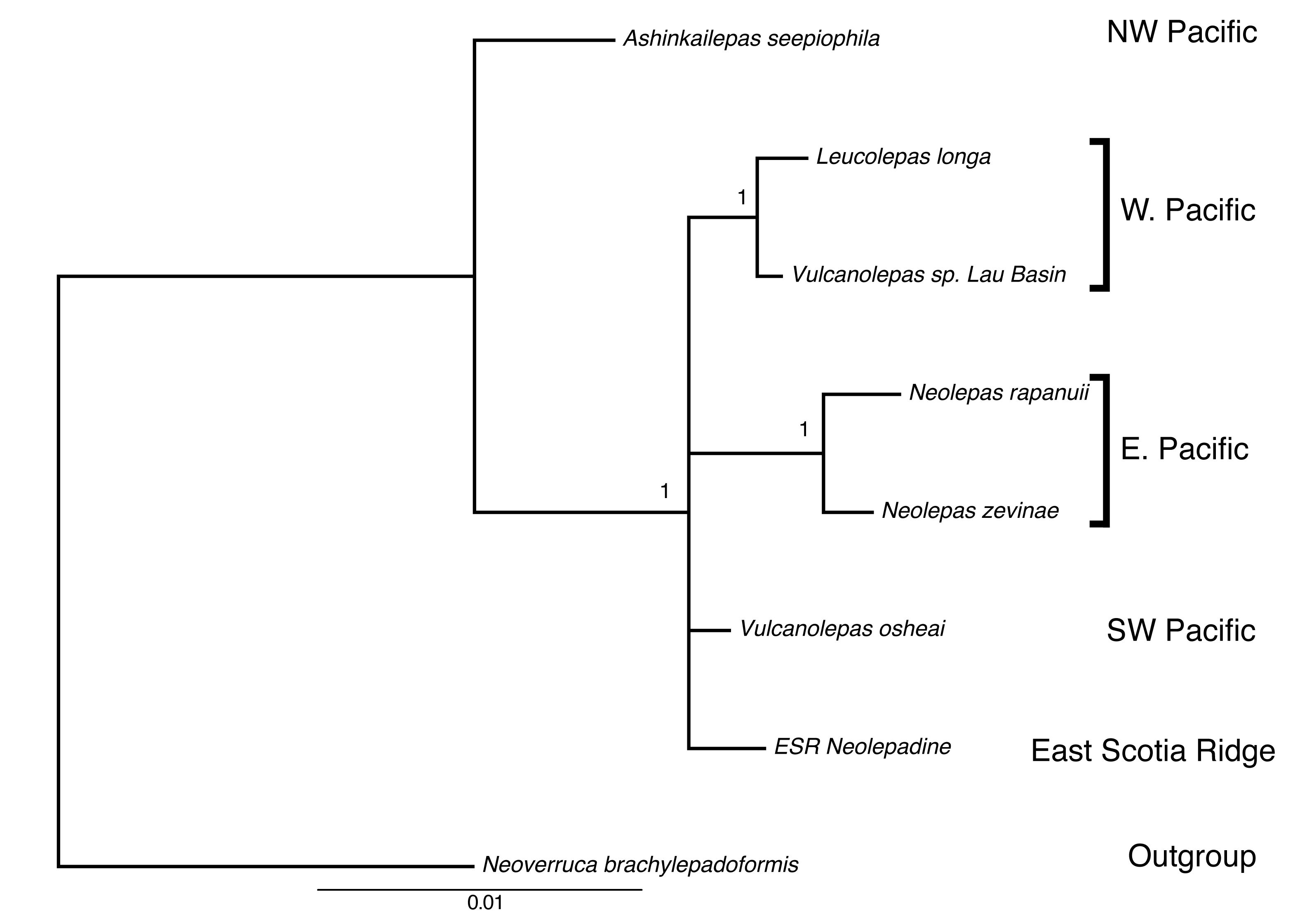

Supplement: Figure S3 — Phylogenetic tree for Vulcanolepas based on histone H3 and 28S rDNA. Phylogenetic tree showing the relationships of stalked barnacles, including Vulcanolepas n. sp., derived from a concatenated sequence of histone H3 and nuclear 28S rDNA gene 1,223 base pairs in length based on Bayesian inference. Values above nodes are Bayesian posterior probability values. Scale bars indicate percent sequence divergence. All nodes with p<0.5 were collapsed into basal polytomies. (TIF) [file pbio.1001234.s003.tif]

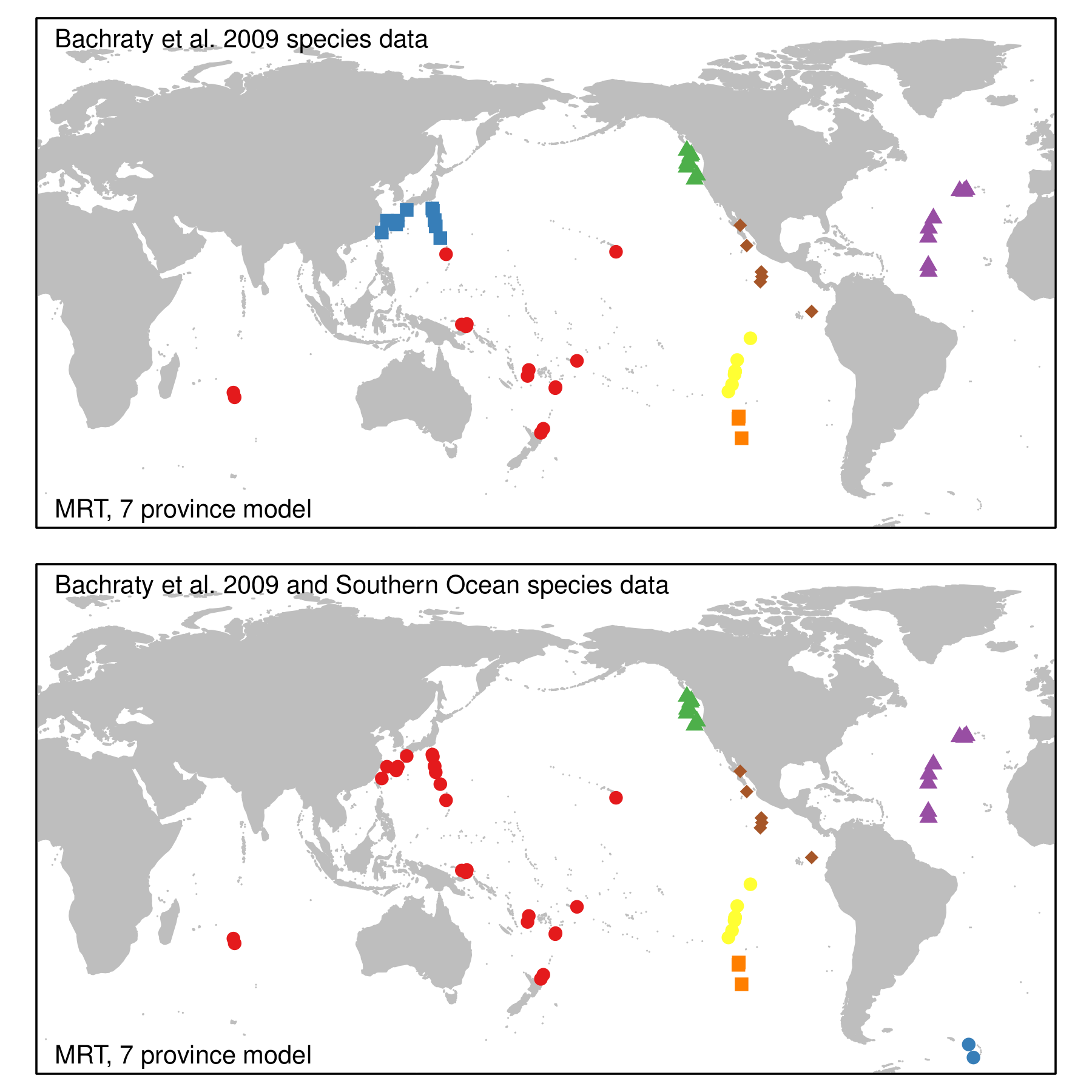

Supplement: Figure S4 — Multivariate regression trees for seven province models using the Bachraty et al. [8] and combined datasets. (A) Results of geographically constrained clustering using MRTs and a seven province model based on the data from Bachraty et al. [8]. This model recovers all provinces proposed by Bachraty et al. [8], with an additional split in the South East Pacific Rise. (B) Results of geographically constrained clustering using MRTs and a seven-province model based on the data from Bachraty et al. [8] and the Southern Ocean sites described in this study. This model does not recover the North West Pacific province proposed by Bachraty et al. [8]; instead, it supports the additional split in the South East Pacific Rise, as well as a separate province for the Southern Ocean sites. (TIF) [file pbio.1001234.s004.tif]

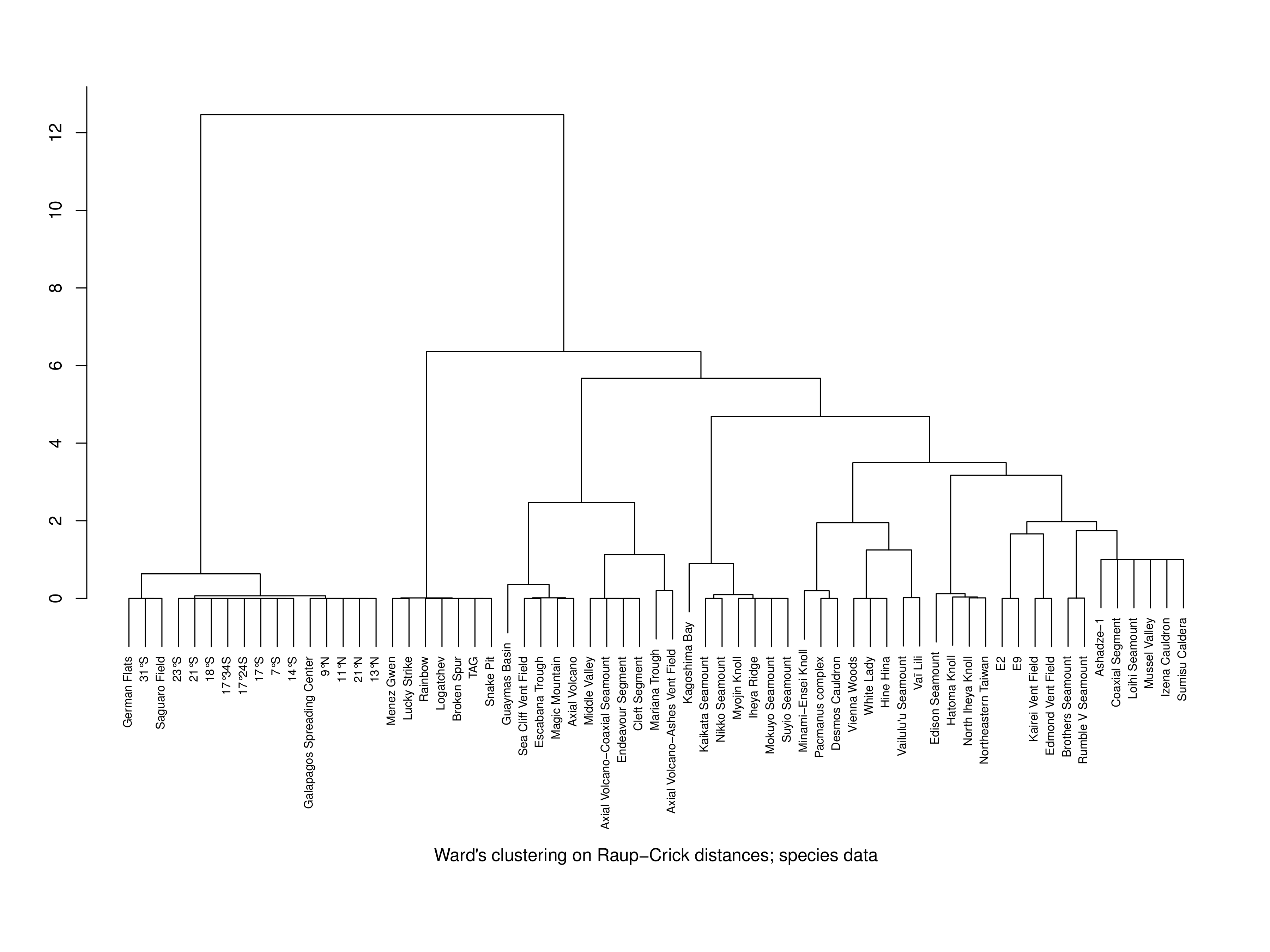

Supplement: Figure S5 — Results of hierarchical agglomerative cluster analysis of community composition data at species level. The tree is based on the Raup-Crick similarity coefficient, a probabilistic measure for presence/absence data. (TIF) [file pbio.1001234.s005.tif]

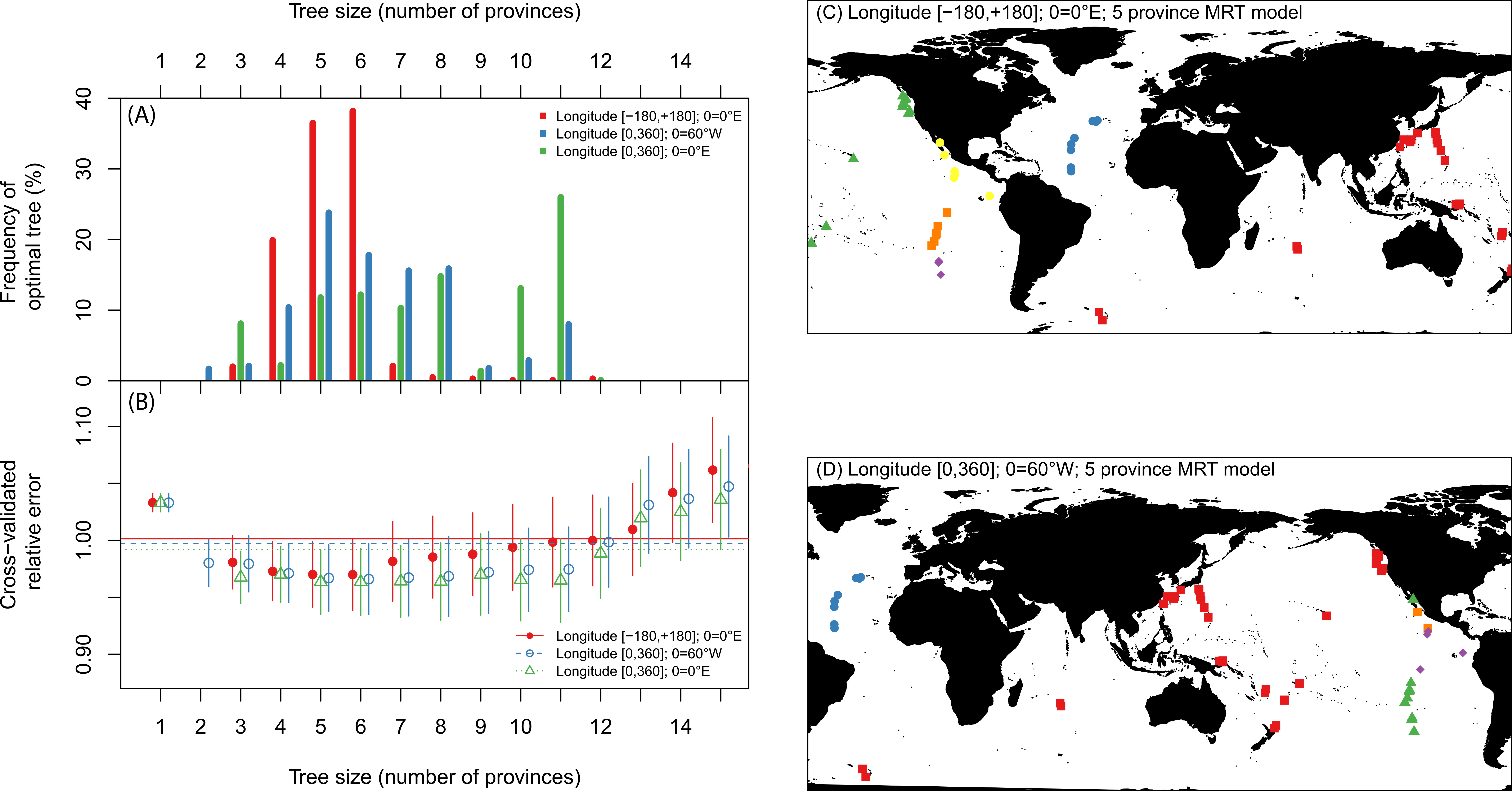

Supplement: Figure S6 — Selection of the multivariate regression tree for a global dataset of vent species using different representations of longitude. The dataset is the species data from Bachraty et al. [8], with Southern Ocean vent sites added (combined dataset). Longitude representations are −180° to +180°, centred on Greenwich (red/filled circles/solid line), 0° to 360° east of 60°W (blue/open circles/dashed line) and 0 to 360° east of Greenwich (green/open triangles/dotted line). (A) Frequency plot of the optimal tree sizes for 1,000 multiple cross-validations. The most common optimal tree size was five and six provinces for the traditional −180° to 180° representation of longitude, five provinces for eastings from 60°W, and 11 provinces for eastings from Greenwich. (B) The cross-validated relative error indicates that predictive power is similar for a wide range of tree sizes. Vertical bars indicate ± one standard error, and the horizontal lines indicate one standard error above the minimum cross-validated relative error. (C and D) Geographic representation of the effects of different longitude encodings. The world map is shifted accordingly to illustrate the edges introduced by using latitude and longitude like Cartesian coordinates. Note the differing provinces in the East Pacific. (C) A five-province model based on the traditional −180° to 180° representation of longitude. (D) A five-province model based on eastings from 60°W. (TIF) [file pbio.1001234.s006.tif]
