# Supplementary material for: The Discovery of New Deep-Sea Hydrothermal Vent Communities in the Southern Ocean and Implications for Biogeography
Source: PLoS Biol. 2012 Jan 3;10(1):e1001234. doi: 10.1371/journal.pbio.1001234 (PMC3250512; doi:10.1371/journal.pbio.1001234)
Supplement: Table S1 — For Kiwa n. sp., primers used for amplification and sequencing of 16S mitochondrial rDNA and 18S and 28S nuclear rDNA genes. (DOC) [file pbio.1001234.s007.doc]

Table S1 *Kiwa* n. sp. Primers used for amplification and sequencing of 16S mitochondrial rDNA and 18S and 28S nuclear rDNA genes.

| **Gene** | **Primer** |  |  | **Reference** |
| --- | --- | --- | --- | --- |
| 16S | 16S L | PCR + Seq Forward | CGCCTGTTTAACAAAAACAT | [87] |
|  | 16S H | PCR + Seq Reverse | CCGGTCTGAACTCAGATCACGT | [87] |
| 18S | 18e | PCR + Seq Forward | CTGGTTGATCCTGCCAGT | [88] |
|  | 18S F448 | Seq Forward | GGAGAGGGAGCCTGAGAAAC | Present study |
|  | 18S F896 | Seq Forward | TTAGAGTGCTCAGAGCAGGC | Present study |
|  | 18S F1437 | Seq Forward | ATGGCCGTTCTTAGTTGGTG | Present study |
|  | 18S F1857 | Seq Forward | TTCCCATGAACGAGGAATTC | Present study |
|  | 18P | PCR + Seq Reverse | TAATGATCCTTCCGCAGGTTCACCT | [88] |
|  | 18S R498 | Seq Reverse | AAGGGCATCACAGACCTGTT | Present study |
|  | 18S R1074 | Seq Reverse | TATCTGATCGCCTTCGAACC | Present study |
|  | 18S R1536 | Seq Reverse | ACGAGCTTTTTAACCGCAAC | Present study |
| 28S | 28S-F216 | PCR + Seq Forward | CTGAATTTAAGCATATTAATTAGKGSAGG | [89] |
|  | 28S-R443 | PCR + Seq Reverse | CCTCACGGTACTTGTTCGCTATCGG | [89] |
