# Supplementary material for: The Discovery of New Deep-Sea Hydrothermal Vent Communities in the Southern Ocean and Implications for Biogeography
Source: PLoS Biol. 2012 Jan 3;10(1):e1001234. doi: 10.1371/journal.pbio.1001234 (PMC3250512; doi:10.1371/journal.pbio.1001234)
Supplement: Table S2 — For Vulcanolepas n. sp., primers used for amplification and sequencing of histone H3 and 28S nuclear rDNA genes. (DOC) [file pbio.1001234.s008.doc]

Table S2 *Vulcanolepas* n. sp. Primers used for amplification and sequencing of histone 3 and 28S nuclear rDNA genes.

| **Gene** | **Primer** |  |  | **Reference** |
| --- | --- | --- | --- | --- |
| H3 | H3 AF | PCR + Seq Forward | ATGGCTCGTACCAAGCAGACVGC | [90] |
|  | H3 AR | PCR + Seq Reverse | ATATCCTTRGGCATRATRGTGAC | [90] |
| 28S | 28S F862 | PCR + Seq Forward | GTCTAACATGTGAGCGAGCG | Present Study |
|  | 28S F1392 | Seq Forward | GGCCACTTTTGGTAAGCAGA | Present Study |
|  | 28S R4 | PCR + Seq Reverse | AACCCATGTTGCTCTGCTTT | Present Study |
|  | 28S R469 | Seq Reverse | TCGTGAGCAATCAACACCTT | Present Study |
|  | 28S R771 | Seq Reverse | ATGGTTCGATTGGTCTTTCG | Present Study |
| **Gene** | **Primer** |  |  | **Reference** |
| H3 | H3 AF | PCR + Seq Forward | ATGGCTCGTACCAAGCAGACVGC | [90] |
|  | H3 AR | PCR + Seq Reverse | ATATCCTTRGGCATRATRGTGAC | [90] |
| 28S | 28S F862 | PCR + Seq Forward | GTCTAACATGTGAGCGAGCG | Present Study |
|  | 28S F1392 | Seq Forward | GGCCACTTTTGGTAAGCAGA | Present Study |
|  | 28S R4 | PCR + Seq Reverse | AACCCATGTTGCTCTGCTTT | Present Study |
|  | 28S R469 | Seq Reverse | TCGTGAGCAATCAACACCTT | Present Study |
|  | 28S R771 | Seq Reverse | ATGGTTCGATTGGTCTTTCG | Present Study |
