# Supplementary material for: The Discovery of New Deep-Sea Hydrothermal Vent Communities in the Southern Ocean and Implications for Biogeography
Source: PLoS Biol. 2012 Jan 3;10(1):e1001234. doi: 10.1371/journal.pbio.1001234 (PMC3250512; doi:10.1371/journal.pbio.1001234)
Supplement: Table S3 — For Lepetodrilus n. sp., primers used for amplification and sequencing of cytochrome oxidase I. (DOC) [file pbio.1001234.s009.doc]

Table S3 *Lepetodrilus* n. sp. Primers used for amplification and sequencing of COI.

| **Gene** | **Primer** |  |  | **Reference** |
| --- | --- | --- | --- | --- |
| COI | LCO1490 | PCR + Seq Forward | GGTCAACAAATCATAAAGATATTGG | [69] |
|  | HCO2198 | PCR + Seq Reverse | TAAACTTCAGGGTGACCAAAAAATCA | [69] |
