# Supplementary material for: The Discovery of New Deep-Sea Hydrothermal Vent Communities in the Southern Ocean and Implications for Biogeography
Source: PLoS Biol. 2012 Jan 3;10(1):e1001234. doi: 10.1371/journal.pbio.1001234 (PMC3250512; doi:10.1371/journal.pbio.1001234)
Supplement: Table S4 — Sequences used for phylogenetic analysis of 16S rDNA to show the relationship of Kiwa n. sp. with other anomuran taxa. (DOC) [file pbio.1001234.s010.doc]

Table S4 Sequences used for phylogenetic analysis of 16S rDNA to show the relationship of *Kiwa* n. sp. with other anomuran taxa.

| Species | GenBank Acc# | Reference |
| --- | --- | --- |
| *Dromia dehaani* | AY583899 | [87] |
| *Uroptychus nitidus* | AY595925 | [87] |
| *Uroptychus parvulus* | AY595926 | [87] |
| *Kiwa hirsuta* | EU831286 | [89] |
| *Kiwa* n. sp. | JN628249 | Present study |
| *Eumunida sternomaculata* | AY351260 | [30] |
| *Pseudomunida fragilis* | EU821536 | [89] |
| *Aegla intercalata* | AY595919 | [68] |
| *Lomis hirta* | AF436052 | [91] |
| *Emerita analoga* | AF425322 | Zaklan & Cunningham, unpublished, 2001 |
| *Blepharipoda occidentalis* | AF436053 | [91] |
| *Lithodes maja* | AF425330 | Zaklan & Cunningham, unpublished, 2001 |
| *Pagurus forceps* | FJ869148 | [92] |
| *Munidopsis recta* | EF428964 | [93] |
| *Shinkaia crosnieri* | EU420129 | [94] |
| *Leiogalathea laevirostris* | AY351252 | [30] |
