# Supplementary material for: The Discovery of New Deep-Sea Hydrothermal Vent Communities in the Southern Ocean and Implications for Biogeography
Source: PLoS Biol. 2012 Jan 3;10(1):e1001234. doi: 10.1371/journal.pbio.1001234 (PMC3250512; doi:10.1371/journal.pbio.1001234)
Supplement: Table S5 — Sequences used for phylogenetic analysis of H3 and 28S rDNA to show the relationship of Vulcanolepas n. sp. with other stalked barnacles from deep-sea hydrothermal vents. (DOC) [file pbio.1001234.s011.doc]

Table S5 Sequences used for phylogenetic analysis of H3 and 28S rDNA to show the relationship of *Vulcanolepas* n. sp. with other stalked barnacles from deep-sea hydrothermal vents.

| Species | H3 GenBack Acc# | 28S GenBank Acc# | Reference |
| --- | --- | --- | --- |
| *Neoverruca brachylepadoformis* | EU082357 | EU082317 | [35] |
| *Ashinkailepas seepiophila* | EU082354 | EU082314 | [35] |
| *Leucolepas longa* | EU082351 | EU082311 | [35] |
| *Neolepas rapanui* | EU082349 | EU082309 | [35] |
| *Neolepas zevinae* | EU082350 | EU082310 | [35] |
| *Vulcanolepas osheai* | EU082353 | EU082313 | [35] |
| *Vulcanolepas* n. sp. (ESR) | JN628252 | JN628523 | Present study |
| *Vulcanolepas* sp. | EU082352 | EU082312 | [35] |
