# Supplementary material for: The Discovery of New Deep-Sea Hydrothermal Vent Communities in the Southern Ocean and Implications for Biogeography
Source: PLoS Biol. 2012 Jan 3;10(1):e1001234. doi: 10.1371/journal.pbio.1001234 (PMC3250512; doi:10.1371/journal.pbio.1001234)
Supplement: Table S6 — Sequences used for phylogenetic analaysis of cytochrome oxidase I to show the relationship of Lepetodrilus n. sp. with other lepetodrilid limpets from deep-sea hydrothermal vents. (DOC) [file pbio.1001234.s012.doc]

Table S6 Sequences used for phylogenetic analaysis of CO1 to show the relationship of *Lepetodrilus* n. sp. with other lepetodrilid limpets from deep-sea hydrothermal vents.

| Species | GenBank Acc# | Reference |
| --- | --- | --- |
| *Pseudorimula* sp. Lau | AB365216 | [95] |
| *Pseudorimula* sp. SBJ-2008 | EU306388 | [33] |
| *Lepetodrilus gordensis* | DQ228050 | [96[ |
| *Lepetodrilus fucensis* | DQ228017 | [96] |
| *Lepetodrilus elevatus* | EU306403 | [33] |
| *Lepetodrilus tevnianus* | EU306391 | [33] |
| *Lepetodrilus cristatus* | EU306428 | [33] |
| *Lepetodrilus ovalis* | EU306484 | [33] |
| *Lepetodrilus pustulosus* | EU306462 | [33] |
| *Lepetodrilus atlanticus* | EU306448 | [33] |
| ESR | JN628254 | Present study |
| *Lepetodrilus* CIR1 | EU306475 | [33] |
| *Lepetodrilus* CIR2 | EU306477 | [33] |
| *Lepetodrilus nux* | EU306444 | [33] |
| *Lepetodrilus schrolli* | EU306442 | [33] |
