# Supplementary material for: The Discovery of New Deep-Sea Hydrothermal Vent Communities in the Southern Ocean and Implications for Biogeography
Source: PLoS Biol. 2012 Jan 3;10(1):e1001234. doi: 10.1371/journal.pbio.1001234 (PMC3250512; doi:10.1371/journal.pbio.1001234)
Supplement: Text S1 — Supplementary information. (DOC) [file pbio.1001234.s013.doc]

**Supplementary Information**

**Methods**

Exploration of the MRT models

Because of the identification of the 6 province model in Bachraty et al. [8] this model was specifically analysed in the present study. In addition, we investigated a 7 province model of vent biogeography, which showed a very similar cross-validation error to a 6 province model but which was more frequently selected as an optimal tree in our analyses for the Bachraty et al. [8] data (Fig. S4 A). A 7 province model is shown for the combined dataset for comparison (Fig. S4 B).

Agglomerative clustering

Geographically unconstrained clustering was performed as a comparison to MRT. Dissimilarity matrices were generated from the combined dataset for species occurrence at hydrothermal vents. These were based on a variety of matrices but results are shown here for Raup-Crick indices only [84] (Fig. S5). This is because this matrix is favoured by the palaeontological community for analyses of datasets comprising presence-absence data only. Dissimilarity matrices were generated using the ‘vegan’ package [85] and hierarchical agglomerative clustering was performed using Ward's minimum variance method in *R* [84].

Assignment of longitude in MRT

Longitude was reset to a 360 ^o^ scheme with 0 ^o^ set at 60 ^o^W or 0 ^o^E and also to a -180 ^o^ and +180 ^o^ scheme with 0 ^o^ set at 0 ^o^E. MRT analyses were repeated as for the main analysis with optimal tree size being investigated by running 1000 multiple cross-validations on each data set (Fig. S6 A-D).

**Results & Discussion**

Exploration of the MRT models

The MRT analyses, with cross-validation, produced a series of trees, many of which were only marginally worse than the best predictive tree (see main paper Fig. 5). This was also the case with the analyses presented by Bachraty et al. [8] but these authors identified a 6 province model as being close to the model with the lowest cross-validation error. In the present analyses instead of a split between the NW Pacific and SW Pacific, indicated in their study, we consistently obtained a six province model with one large contiguous Indo-Pacific province but with a split along the Southern East-Pacific Rise province. The NW Pacific split, however, was retained in a seven province model of their data (Fig. S4 A). When adding species data for the Southern Ocean sites, a seven province model again favours a contiguous Indo-Pacific province, while classifying the Southern Ocean sites as a separate cluster (Fig. S4 B). As with the 6-province model, both the 7-province analyses and the optimal 11- province analyses with the Southern Ocean vent fauna, the sites south of the Easter Microplate, in the South East Pacific, form a separate cluster from all other East Pacific sites.

Agglomerative clustering

The results for agglomerative clustering are shown in Figure S5. The initial split in the agglomerative tree is between the eastern Pacific sites and all other hydrothermal vents. Sites south of the Easter Microplate form a separate cluster within the eastern Pacific subset of vents. Within the second group of hydrothermal vents, comprising all other sites, the Atlantic vents, with the exception of Ashadze-1, form a distinct cluster. The North East Pacific sites including all those on the Gorda, Juan de Fuca, and Explorer Ridges all form a single cluster. The western Pacific sites form the remaining clusters, with the Indian Ocean, East Scotia Ridge and Kermadec Ridge sites all forming separate clusters nested within the larger western Pacific set. The results of the agglomerative clustering using the Raup-Crick dissimilarity matrix are most similar to the favoured optimum MRT analysis for the combined dataset with the obvious exception being the Ashadze-1 vent field. This hydrothermal vent system is unusual in that it has no megafauna that host chemosymbiotic bacteria but a fauna typically associated with the periphery of vent fields in the Atlantic Ocean [86]. The site may represent the declining stages of hydrothermal activity [86].

Assignment of longitude

Resetting the scheme of longitudinal assignment markedly changed both the optimal tree size (S6 A,B) and the clustering results (S6 C, D). This suggests that the encoding of geographical coordinates and their treatment as Cartesian coordinates in this form of analysis has a profound influence on clustering results.

**References**

1. Legendre P and Legendre L (1998) Numerical Ecology. 2^nd^ English Edition. Elsevier Science B.V., Amsterdam, 853 pp.
2. Oksanen J, Blanchet FG, Kindt R, Legendre P, O'Hara RB et al. (2011) vegan: Community Ecology Package. R package version 1.17-12. <http://CRAN.R-project.org/package=vegan>
3. Fabri M-C, Bargain A, Briand P, Gebruk A, Fouquet Y et al. (2011) The hydrothermal vent community of a new deep-sea field, Ashadze-1, 12^o^58′N on the Mid-Atlantic Ridge. J Mar Biol Ass UK 91:1-13.
4. Ahyong ST, O‘Meally D (2004) Phylogeny of the Decapoda Reptantia: Resolution using three molecular loci and morphology. The Raffles Bull Zool 52: 673–693.
5. Halanych KM, Lutz RA, Vrijenhoek RC (1998) Evolutionary origins and age of vestimentiferan tube worms. Cah Biol Mar 39: 355–358.
6. Ahyong ST, Schnabel KE, Maas EW (2009) Anomuran phylogeny: new insights from molecular data. In: Martin JW, Felder DL, Crandall KA (Eds), Decapod Crustacean Phylogenetics Crustacean Issues 18 (CRC Press, Bocan Raton, FL), 391–408.
7. Colgan DJ, McLauchlan A, Wilson GDF, Livingston SP, Edgecombe GD et al. (1998) Molecular phylogenetics of the Arthropoda: relationships based on Histone H3 and U2 snRNA DNA. Australian J Zool 46: 419–437.
8. Morrison CL, Harvey AW, Lavery S, Tieu K, Huang Y et al. (2002) Mitochondrial gene rearrangements confirm the parallel evolution of the crab- like form. Proc Roy Soc Lond B 269: 345-350.
9. Mantelatto FL, Pardo LM, Pileggi LG, Felder DL (2009) Taxonomic re-examination of the hermit crab species *Pagurus forceps* and *Pagurus comptus* (Decapoda: Paguridae) by molecular analysis. Zootaxa, 2133: 20–31.
10. Terrat Y, Bonnivard E, Higuet D (2008) GalEa retrotransposons from galatheid squat lobsters (Decapoda, Anomura) define a new clade of Ty1/copia-like elements restricted to aquatic species. Mol Genet Genomics 279:63-73.
11. Yang JS, Nagasawa H, Fujiwara Y, Tsuchida S, Yang WJ (2008) The complete mitochondrial genome sequence of the hydrothermal vent galatheid crab *Shinkaia crosnieri* (Crustacea: Decapoda: Anomura): a novel arrangement and incomplete tRNA suite. BMC Genomics 9:257. doi:10.1186/1471-2164-9-257
12. Kano Y (2008) Vetigastropod phylogeny and a new concept of Seguenzioidea: independent evolution of copulatory organs in the deep-sea habitat. Zool Scripta 37: 1–21.
13. Johnson SB, Young CR, Jones WJ, Warén A, Vrijenhoek RC (2006) Migration, isolation and speciation of hydrothermal vent limpets (Gastropoda: Lepetodrilidae) across the Blanco Transformation Fault. Biol Bull 210: 140–157.
